# Roadmap on Recent Progress in FINCH Technology

**DOI:** 10.3390/jimaging7100197

**Published:** 2021-09-29

**Authors:** Joseph Rosen, Simon Alford, Vijayakumar Anand, Jonathan Art, Petr Bouchal, Zdeněk Bouchal, Munkh-Uchral Erdenebat, Lingling Huang, Ayumi Ishii, Saulius Juodkazis, Nam Kim, Peter Kner, Takako Koujin, Yuichi Kozawa, Dong Liang, Jun Liu, Christopher Mann, Abhijit Marar, Atsushi Matsuda, Teruyoshi Nobukawa, Takanori Nomura, Ryutaro Oi, Mariana Potcoava, Tatsuki Tahara, Bang Le Thanh, Hongqiang Zhou

**Affiliations:** 1 School of Electrical and Computer Engineering, Ben-Gurion University of the Negev, P.O. Box 653, Beer-Sheva 8410501, Israel; 2 Department of Anatomy and Cell Biology, University of Illinois at Chicago, 808 South Wood Street, Chicago, IL 60612, USA; sta@uic.edu (S.A.); jart@uic.edu (J.A.); mpotcoav@uic.edu (M.P.); 3 Optical Sciences Centre and ARC Training Centre in Surface Engineering for Advanced Materials (SEAM), School of Science, Swinburne University of Technology, Hawthorn, VIC 3122, Australia; vanand@swin.edu.au (V.A.); sjuodkazis@swin.edu.au (S.J.); 4 Institute of Physical Engineering, Faculty of Mechanical Engineering, Brno University of Technology, Tech-nická 2, 619 69 Brno, Czech Republic; petr.bouchal@ceitec.vutbr.cz; 5 Central European Institute of Technology, Brno University of Technology, Purkyňova 656/123, 612 00 Brno, Czech Republic; 6 Department of Optics, Palacký University, 17. Listopadu 1192/12, 771 46 Olomouc, Czech Republic; bouchal@optics.upol.cz; 7 School of Information and Communication Engineering, Chungbuk National University, 1 Chungdae-ro, Seowon-gu, Cheongju 28644, Chungbuk, Korea; uchka@chungbuk.ac.kr (M.-U.E.); namkim@chungbuk.ac.kr (N.K.); 8 Beijing Engineering Research Center of Mixed Reality and Advanced Display, School of Optics and Photonics, Beijing Institute of Technology, Beijing 100081, China; huanglingling@bit.edu.cn (L.H.); hzhou@bit.edu.cn (H.Z.); 9 Faculty of Life & Environmental Sciences, Teikyo University of Science, 2525 Yatsuzawa, Uenohara, Yamanashi 409-0193, Japan; ayumi-i@ntu.ac.jp; 10 PRESTO, Japan Science and Technology Agency, 4-1-8 Honcho, Kawaguchi 332-0012, Saitama, Japan; 11 Tokyo Tech World Research Hub Initiative (WRHI), School of Materials and Chemical Technology, Tokyo Institute of Technology, 2-12-1, Ookayama, Meguro-ku, Tokyo 152-8550, Japan; 12 School of Electrical and Computer Engineering, University of Georgia, Athens, GA 30602, USA; kner@uga.edu; 13 Advanced ICT Research Institute Kobe, National Institute of Information and Communications Technology, 588-2 Iwaoka, Iwaoka-cho, Nishi-ku, Kobe 651-2492, Japan; koujin0821@gmail.com (T.K.); a.matsuda@nict.go.jp (A.M.); 14 Institute of Multidisciplinary Research for Advanced Materials, Tohoku University, 2-1-1 Katahira, Aoba-ku, Sendai 980-8577, Japan; y.kozawa@tohoku.ac.jp; 15 School of Physics Science and Engineering, Tongji University, Shanghai 200092, China; liangdong@siom.ac.cn; 16 State Key Laboratory of High Field Laser Physics, Shanghai Institute of Optics and Fine Mechanics, Chinese Academy of Sciences, Shanghai 201800, China; jliu@siom.ac.cn; 17 Center of Materials Science and Optoelectronics Engineering, University of Chinese Academy of Sciences, Beijing 100049, China; 18 Department of Applied Physics and Materials Science, Northern Arizona University, Flagstaff, AZ 86011, USA; Christopher.Mann@nau.edu; 19 Center for Materials Interfaces in Research and Development, Northern Arizona University, Flagstaff, AZ 86011, USA; 20 Wallace H. Coulter Department of Biomedical Engineering, Georgia Institute of Technology and Emory University, Atlanta, GA 30332, USA; abhijit.marar@gmail.com; 21 Science & Technology Research Laboratories, Japan Broadcasting Corporation (NHK), 1-10-11 Kinuta, Setagaya-ku, Tokyo 157-8510, Japan; nobukawa.t-eq@nhk.or.jp; 22 Faculty of Systems Engineering, Wakayama University, Sakaedani 930, Wakayama 640-8510, Japan; nom@wakayama-u.ac.jp; 23 Applied Electromagnetic Research Center, Radio Research Institute, National Institute of Information and Communications Technology (NICT), 4-2-1 Nukuikitamachi, Koganei, Tokyo 184-8795, Japan; oi.ryutaro@nict.go.jp (R.O.); tahara@nict.go.jp (T.T.); 24 Viettel High Technology Industries Corporation, 380 Lac Long Quan Street, Hoan Kiem, Hanoi 100000, Vietnam; banglt2@viettel.com.vn

**Keywords:** Fresnel incoherent correlation holography, incoherent holography, digital holography, color holography, digital holographic microscopy, computational coherent superposition, phase-shifting interferometry, multiplexed imaging, fluorescence microscopy, lattice light-sheet holography, single-molecule localization microscopy, metasurfaces

## Abstract

Fresnel incoherent correlation holography (FINCH) was a milestone in incoherent holography. In this roadmap, two pathways, namely the development of FINCH and applications of FINCH explored by many prominent research groups, are discussed. The current state-of-the-art FINCH technology, challenges, and future perspectives of FINCH technology as recognized by a diverse group of researchers contributing to different facets of research in FINCH have been presented.

## 1. Introduction (Joseph Rosen and Vijayakumar Anand)

Fresnel incoherent correlation holography (FINCH) is a method of recording incoherent digital Fresnel hologram. This roadmap article provides an overview of research activities in the technology of FINCH by several prominent researchers in the field. FINCH was proposed in 2007 [1] as a method of recording incoherent digital holograms without scanning. FINCH was inspired by several previous methods and systems [2,3,4,5,6] and has inspired many studies since then; some of them are described in the following sections of this article. In this introduction, we choose to open the roadmap article with a summary of the technological development of FINCH and other similar systems from the perspective of those who have been following the field since its birth.

**Figure 1:**
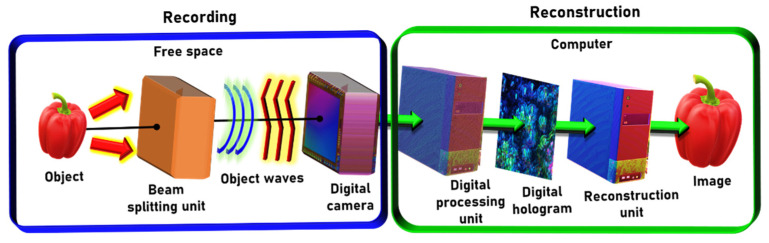
Recording and reconstruction of a hologram in a general self-interference digital holography system.

FINCH belongs to the large family of self-interference digital holography systems [7]. The general optical configuration of these systems is shown in Figure 1. The light emitted from each object point is collected by a beam splitting unit, in which the input wave is split into two, whereas each wave is modulated differently. Since the two waves are originated from the same object point, they are mutually coherent, and hence they can interfere on the digital camera plane. The presence of object information in both interfering waves introduces peculiar imaging characteristics such as violating the Lagrange invariant [8]. The digital camera accumulates the entire interference patterns of all the object points to an incoherent hologram. A single hologram, or several acquired holograms, are fed inside a digital computer. In the case of several holograms, they are superposed into a single digital hologram. Finally, the three-dimensional (3D) image of the object is reconstructed in the computer from the processed hologram by a numerical process called Fresnel backpropagation [9]. Fresnel backpropagation is a two-dimensional convolution of the hologram with a quadratic phase function, where the *z* distance between the hologram and the reconstructed plane is a parameter in the quadratic phase function.

Several aspects of the above description of FINCH have been implemented in many ways. For example, beam splitting and different beam modulation were originally performed by spatial multiplexing of two different diffractive optical elements on the same single optical channel [1]. Another method of multiplexing two different diffractive optical elements on one optical channel is termed polarization multiplexing [10]. In this method, the light propagates in two orthogonal polarizations, each of which is modulated differently by at least one of the optical devices of the system. Other researchers proposed using a glassy beamsplitter and two different interferometer channels for the same purpose [11]. Another aspect of FINCH is the number of camera shots needed for reconstructing the observed scene. To remove the unnecessary images known as the twin image and the bias term, some researchers use the phase-shifting procedure implemented on three [1] or four [12] camera shots. Other researchers use a single camera shot and different ways to eliminate the twin image and the bias term [13,14,15,16].

Together with the structural evolution of FINCH, the diversity of applications using these systems is growing rapidly. The common application for digital holography is the 3D imaging of incoherently illuminated objects shown in the first demonstration of FINCH [1]. A short time after, the 3D imaging was extended to fluorescence microscopy [17], which was later demonstrated in FINCH systems with transmission liquid crystal GRIN lens [18]. In the same world of optical microscopy, there are two other applications that appear with structural modifications over the original FINCH. One of them is microscopy with axial sectioning demonstrated with two successive spatial light modulators (SLMs)—one is used to modulate the two beams differently, and the other is positioned at the front image plane for sectioning [19]. A closely related technique called confocal incoherent correlation holography was demonstrated using a spinning disk [20]. Other microscopy-related techniques are the super-resolution methods. FINCH with a synthetic aperture [21] and structured illumination in FINCH [22] are examples of methods adopted from conventional imaging to improve the resolution of FINCH-based microscopes. A super-resolution technique invented for FINCH is the method with a scattering mask between the observed objects and the hologram recorder [23]. An important additional issue investigated by several research groups is FINCH and similar incoherent hologram recorders operating with polychromatic light [24,25,26].

Fourier incoherent single-channel holography (FISCH) keeps the principle of FINCH of operating with a single coaxial, optical channel [27]. FISCH is implemented by a different type of the general self-interference incoherent interferometer shown in Figure 1. The light emitted from every object point is also split into two mutually coherent beams, and as usual, each of these light beams is modulated differently. However, the main difference between FISCH and FINCH is that in FISCH, the two replications of each object point are imaged to the same transverse plane. Furthermore, one image point is rotated by 180° to the other point, where the rotation axis is at the origin of the plane. The overall recorded hologram is the sum of the entire interference patterns, each of which is linear interference grating created from pair of image points. The image of the object is reconstructed in the computer by a digital 2D Fourier transform.

The first version of FISCH published in 2012 was implemented by passing twice through a single SLM [27]. A year later, a different FISCH setup with two separated SLMs was proposed to reduce the optical path difference between the two interferometer channels [28]. The main motivation for working with FISCH is the theoretical ability to reconstruct the image from a hologram captured by a single camera shot. This ability to separate the undesired terms from the desired image is based on the inherent feature of the Fourier hologram to reconstruct each image and term in a different diffraction order. However, because of the unideal behavior of the SLM, the undesired zero diffraction order shines a bright light on the desired image at the first diffraction order. Consequently, an additional camera shot with different phase constants between the two replicated images was needed to eliminate the zero order from the reconstructed pattern. FINCH and FISCH have inspired other SLM-based incoherent digital holography systems, such as coded aperture correlation holography (COACH) [29].

The research and developments in FINCH followed three paths: super-resolution, development, and applications. The super-resolution of FINCH was investigated in many studies [8,30,31]. The second path is mainly focused on obtaining the optimal architecture and achieving the best performance of FINCH. The third path is on novel applications of FINCH and integrating FINCH with other imaging technologies. In this roadmap, the innovations made by different research groups on the second and third paths, namely optical architectures, beam modulation mechanisms, and reconstruction approaches, are discussed.

After the invention of FINCH in 2007, the implementation went through several stages of upgrading from a spatial random multiplexing approach [1], dual polarizers [10], and a reduced path difference approach using dual diffractive lenses [32]. In 2017, Tahara et al. proposed a radical approach called polarization multiplexing scheme involving the use of a 4-polarization camera and simultaneously recording four camera images in a single shot followed by a computational interpolation and reconstruction method [13]. Nobukawa et al. proposed a smart spatial multiplexing approach using a dual-focus checkerboard lens using the conventional Michelson interferometer-based configuration with two spatial light modulators (SLMs), one for each phase modulation, and achieved single-shot FINCH [33]. Later, in 2020, the research group led by Nomura demonstrated an advanced version of single-shot FINCH by spatial random multiplexing of the two different phase functions and with a single SLM and in a single-channel configuration [34]. In the same year, a geometric phase lens and 4-polarization camera were used to demonstrate single-shot FINCH [35]. Recently, Anand et al. proposed a pattern recognition approach of FINCH in which the point spread hologram (PSH) of FINCH was recorded and cross-correlated with the object hologram. In this study, the beam modulator was randomly multiplexed Fresnel zone lenses fabricated using electron beam lithography [14,36]. In 2020, FINCH was demonstrated for the first time using flat bifocal metalens by Zhou et al. [37].

The third path consists of novel applications of FINCH. In 2012, Bouchal and Bouchal proposed and demonstrated spiral FINCH for selective edge enhancement of observed objects [38]. In this study, one of the object waves was modulated by a spiral quadratic phase function and interfered with the collimated object wave. The hologram during reconstruction generated edge-enhanced version of the object function. This is a potential approach for fluorescence microscopy. In 2014, a holographic method was applied for live-cell 3D imaging and autostereoscopic display by the research group of Kim [39]. Marar and Kner successfully demonstrated single-molecule localization microscopy (SMLM) with FINCH on particles emitting as low as 13,000 photons and can be used for long axial ranges [40]. In 2021, Potcoava et al. demonstrated FINCH in a lattice light sheet [41]. Most recently, Tahara et al. have successfully upgraded FINCH to image in four dimensions (4D) using wavelength multiplexing approach and computational coherent superposition [42]. The interested reader can find more about self-interference digital holography systems in general and about FINCH technology in particular in the following sections. We hope that the sections in this article can be an inspiration in developing the next generation of self-interference digital holography technology.

## 2. Single-Shot Phase-Shifting Fresnel Incoherent Correlation Holography with Dual-Phase Gratings (Teruyoshi Nobukawa)

### 2.1. Status

**Figure 2:**
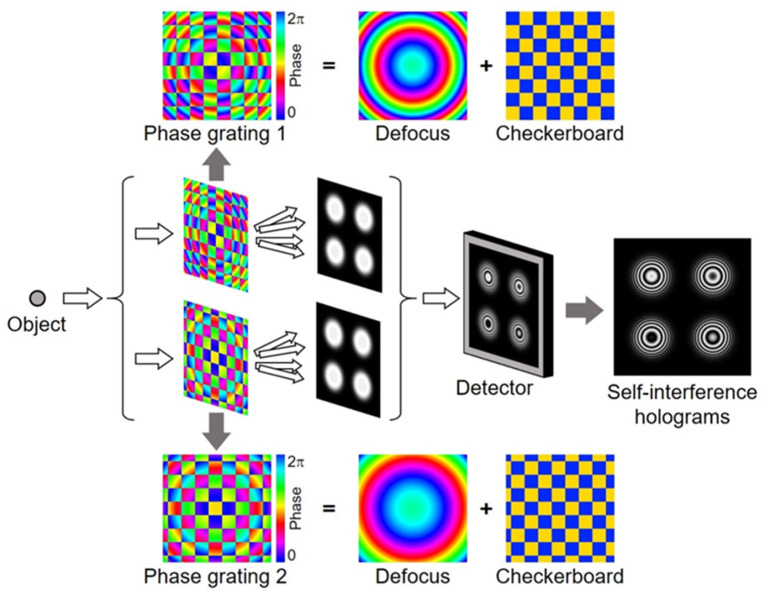
Schematic of single-phot phase-shifting FINCH with dual-phase gratings.

**Figure 3:**
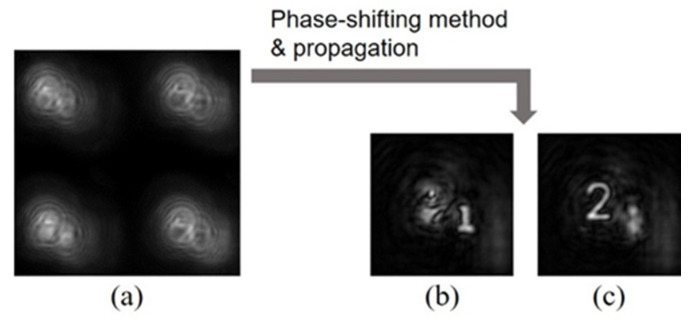
Proof-of-principle experimental results. (**a**) Captured raw image. (**b**,**c**) Reconstructed images through a phase-shifting method and numerical propagation based on an angular spectrum method.

FINCH is an attractive 3D imaging technique because it can create holograms using a spatially incoherent light source [7]. FINCH generally requires a phase-shifting method that records a series of holograms with different phase shifts to eliminate background and conjugate terms. To record the holograms of static objects, a temporal phase-shifting method is used. However, it becomes impractical for capturing moving objects. To record a 3D video, a single-shot technique is required. Figure 2 shows a schematic of one of the phase-shifting techniques for FINCH. The method makes use of dual-phase gratings for creating four holograms in parallel [33]. The dual-phase gratings can be created with a spatial light modulator or a diffracting optical element. The light propagated from an object to be captured is divided into two lights. Each of the lights is modulated with a phase grating, and each of phase gratings consists of defocus and checkerboard phase patterns. The defocus phase pattern encodes the 3D information onto self-reference holograms [7]. The checkerboard phase pattern divides an incident light into four copies, which is necessary to create four holograms. Moreover, the checkerboard phase patterns in each phase grating are laterally displaced with respect to one another, which introduces different phase shifts into four copies on the basis of the Fourier shift theorem. After modulating the lights with the dual-phase gratings, they are recombined for interference. This results in the creation of four self-interference holograms with different phase shifts on an image sensor. Thus, it is possible to capture multiple holograms at the same time and implement the phase-shifting method. Figure 3 shows experimental results [33]. The objects to be captured were two transmission patterns with “1” and “2” on a United State Air Force (USAF) test chart. Figure 3a shows a raw image captured with an image sensor. The four holograms with different phase shifts were successfully created. The holograms were cropped with correlation and subsequently processed based on a phase-shifting algorithm. By applying an angular spectrum method, it is possible to reconstruct images at arbitrary depth planes. Figure 3b,c show the reconstructed images. The objects were successfully reconstructed by the single-shot technique with the dual-phase gratings. The advantage of this method is providing flexible choices for image sensors ranging from standard ones to scientific ones depending on cost, robustness to noise, frame rate, pixel pitch, quantization level, and the number of pixels.

### 2.2. Future Challenges

FINCH requires a temporal coherence to create holograms with high spatial resolution [43]. To enhance the temporal coherence, a narrow bandpass filter is generally applied, which reduces the light utilization efficiency and creates a tradeoff with spatial resolution. Moreover, the single-shot technique using dual-phase gratings divides an incident light into four copies. This also causes a reduction in the light energy of each hologram [33], which seriously affects the framerate of a movie and leads to degradation in image quality due to the noise in the image sensor. Thus, one of the challenges is improving light utilization efficiency. Theoretical investigations suggest that there are potentially better choices for an optical setup that can avoid the tradeoff depending on the depth position of an object [44]. By further exploring the operation of FINCH in detail, optimizing the optical setup for improving light utilization efficiency without loss of spatial resolution becomes necessary. In addition to such analog-based approach, the introduction of a digital-based approach is significant. Even if the light utilization efficiency is reduced by enhancing the framerate or shortening exposure time, if the noise is adequately reduced by digital postprocessing, the FINCH system would work well for capturing 3D movies. Spatial averaging, or finite impulse response filters, is a simple yet powerful tool to effectively reduce noise. By judiciously choosing the filter conditions in spatial averaging, it is possible to reduce noise without loss of spatial resolution [45]. Moreover, recently emerging computer science technologies such as compressive sensing and deep learning would improve the performance of FINCH with an accurate forward model of an optical system [12].

### 2.3. Conclusions

FINCH has expanded the application of holography due to the elimination of the coherence requirement for a light source. One significant application of FINCH is passive 3D imaging under ambient light outdoors. To implement a snapshot of 3D images or record 3D movies, a single-shot technique is promising. Although many single-shot techniques have been proposed [13,34,46,47,48,49,50,51], utilizing dual-phase gratings works with any image sensor in principle, which is useful for optimizing an optical setup with respect to cost, robustness to noise, frame rate, pixel pitch, quantization level, and the number of pixels. By improving light utilization efficiency and reducing the noise, FINCH would be a significant technology for the imaging and measurement community.

## 3. Parallel Phase-Shifting Single-Shot in-Line Fresnel Incoherent Correlation Holography Using a Dual-Focus Checkerboard Lens (Takanori Nomura)

### 3.1. Status

As Fresnel incoherent correlation holography (FINCH) generates twin and zero-order images inherent to holography, it causes a deterioration of reconstructed images. Particularly for an on-axis setup, it is significant. Time-division phase-shifting techniques are good solutions. However, it is not suitable to record dynamic phenomena because the techniques require more than one exposure, typically four. Some methods [33,47,52] have been proposed to realize dynamic recording. A method of obtaining incoherent off-axis holograms by a single-shot off-axis-based FINCH has been proposed [47,52]. It realizes single-shot recording, but the spatial resolution of the hologram is reduced to about one-third. Another method using two diffraction optical elements (DOEs) has been proposed [34]. Although the spatial resolution of the method is half that of the FINCH with the time-division phase-shifting technique, four holograms are obtained simultaneously. In other words, this method has a higher spatial resolution for a hologram than the off-axis-based FINCH. Unfortunately, the optical setup is complicated, and it is difficult to align. Here, an alternative solution of parallel phase-shifting method inspired by [33] is introduced.

**Figure 4:**
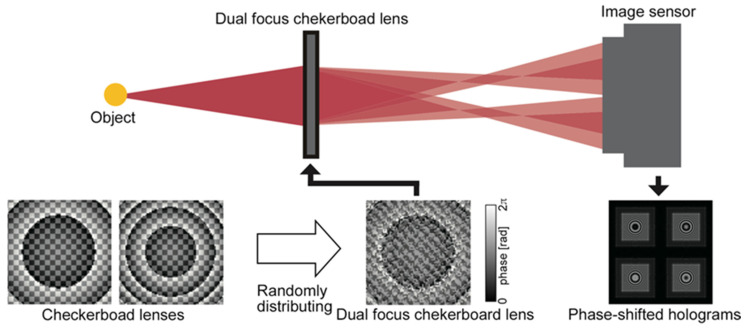
FINCH setup using a dual-focus checkerboard lens for parallel phase shifting.

The optical setup for parallel phase-shifting single-shot in-line Fresnel incoherent correlation holography using a dual-focus checkerboard lens (DFCL) is shown in Figure 4. The details of the design method of DFCL are referred to [34]. The point is that a single DOE, which is a DFCL generates four phase-shifted holograms simultaneously. Therefore, the optical setup is simpler than the others and a single-shot phase-shifting method is realized. In Figure 4, a point source is assumed as an object. You can see phase-shifted Fresnel zone patterns as holograms.

**Figure 5:**
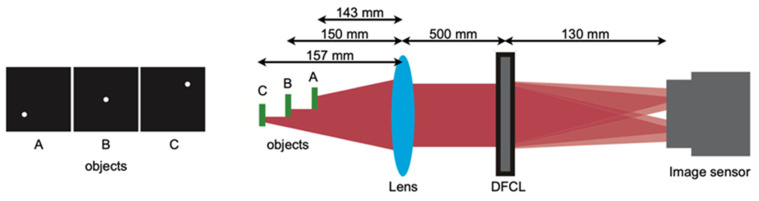
An assumed optical setup for numerical simulations.

To confirm the FINCH using DFCL, numerical simulations were performed. The assumed optical setup is shown in Figure 5. Three spatially incoherent small objects with a line spectrum of a wavelength a 671 nm were assumed as the object. They were placed at distances of 143, 150, and 157 mm, respectively. The two focal lengths of DFCL were infinity and 280 mm. The DOE (DFCL) has 1280 × 1280 pixels, and the size of the pixel is 3.74 mm × 3.74 mm. The image sensor has the same number of pixels and size of pixel as the DOE. Other parameters were shown in Figure 5.

**Figure 6:**
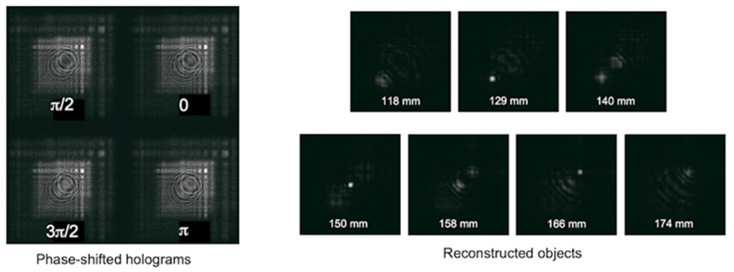
Obtained four phase-shifted holograms and reconstructed images at different axial positions.

Simulation results were shown in Figure 6. Using four phase-shifted holograms (phase shifting quantities are shown in the figure) shown in Figure 6, reconstructed images at different axial distances were obtained. For the conditions in this simulation, the reconstruction distances of the objects were corresponding to 129, 150, and 166 mm, respectively. You can see three small objects were reconstructed at proper distances without a twin and zero-order images.

### 3.2. Conclusions

Parallel phase-shifting single-shot in-line FINCH using a DFCL was described. It makes it possible to record a dynamic phenomenon owing to a single-shot exposure. In addition, its optical setup is very simple due to a DFCL. Numerical simulation results were given to confirm the method. More numerical/experimental results can be obtained from the literature [34].

## 4. Single-Shot Fresnel Incoherent Digital Holography Based on Geometric Phase Lens (Dong Liang and Jun Liu)

### 4.1. Status

Incoherent digital holography techniques connect the research areas digital holography to incoherent optics opening the possibilities applying digital holography principles to fluorescence microscopy and white light imaging [2,53]. Among many optical architectures developed for implementing incoherent holography, FINCH has been one of the widely used configuration as it is non-scanning and motionless [1]. In FINCH, the light from a point is split into two mutually coherent spherical waves with different radii of curvatures and interfered to encode the 3D location of the point. In FINCH, the beam splitting and beam modulation is achieved using an SLM. Many versions of beam splitting configurations were proposed [13,33,34,47,54]. However, the use of SLM comes with certain limitations such as increased complexity of optical configuration and a high cost.

**Figure 7:**
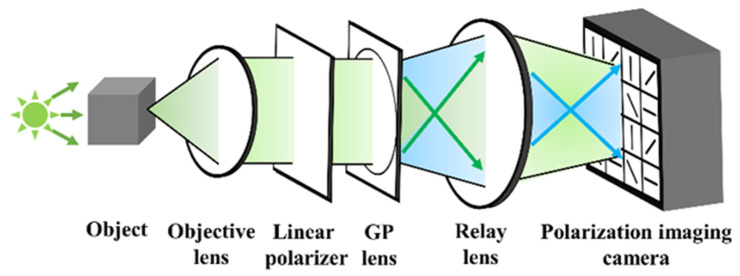
Schematic diagram of the single-shot FINCH system using a GP lens (adapted from [35]).

In this paper, FINCH was demonstrated using a geometric phase (GP) lens [55] with a polarization-dependent focus. The GP lens is based on engineering the Pancharatnam–Berry phase [56] using photo-aligned layers of liquid crystals. The GP lens converts left-hand circular polarization (LHCP) light and right-hand circular polarization (RHCP) light into converging and diverging waves with the same radii of curvatures, respectively. So for a linearly polarized input light, the GP generates both converging and diverging wavefronts with a splitting ratio of 50:50. The GP also offers a wavelength independent efficiency in the range 450–650 nm. The application of GP to FINCH will substantially reduce the cost of implementation of FINCH in a reduced space and offer polarization-sensitive behaviors. Single-shot FINCH has been demonstrated using a 4-polarization camera and with space-division multiplexing [57], as shown in Figure 7.

An object was illuminated by an incoherent light source and the light diffracted from the object was collected and collimated by a convex lens (100 mm). The collimated light was linearly polarized and is incident on a GP lens (#34-466, Edmund optics,100 mm). The GP lens splits the incoming object wave into two: converging and diverging object waves and relayed onto the 4-polarization camera (PHX050S-P, LUCID, 2448 × 2048, pixel pitch = 3.45 µm) using a relay lens. The relay lens is a crucial part of the setup with GP lens as the radii of curvatures of the two interfering beams are large and the overlapped area of the two beams is larger than the sensor area. The above condition also affects the fringe visibility. The 4-polarization camera consists of super pixels where every pixel consists of linear polarizers oriented at 0, π/4, π/2 and 3π/4 in a 2 × 2 formation. So, every single camera shot recorded by this camera is equivalent to four camera shots recorded with different polarizations. The recorded intensity distributions were processed by de-mosaicing and linear interpolation to obtain the missing pixel information. Then, the four intensity distributions were projected into the complex space with the corresponding phase shifts and combined. The resulting complex hologram is numerically propagated to one of the two focal planes of the negative and positive lens of the GP to reconstruct the object information.

**Figure 8:**
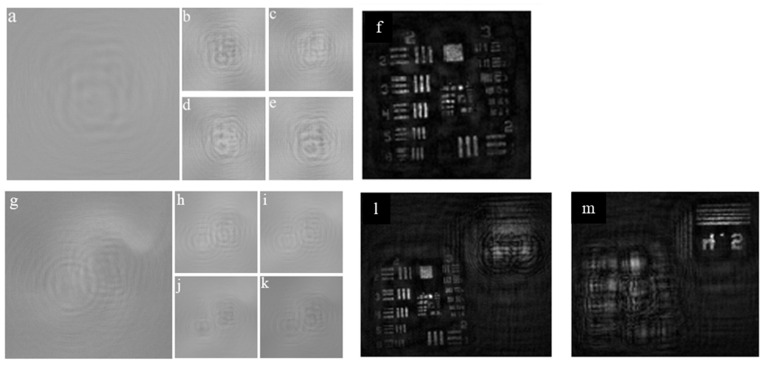
(**a**) Image of the single-shot raw hologram of a single-plane object and (**b**–**e**) four phase-shifted holograms 0, π/4, π/2 and 3π/4, respectively. (**f**) Reconstruction result of a USAF1951 resolution target. (**g**) Image of the single-shot raw hologram of a two-planes object consisting of a USAF1951 object and NBS object separated by a distance of 65 mm, (**h**–**k**) four phase-shifted holograms 0, π/4, π/2 and 3π/4, respectively. Reconstruction result by numerical propagation to the plane of (**l**) USAF and (**m**) NBS. (Adapted from [35].)

The experiment was carried out using USAF and (National Bureau of Standards) NBS objects. In the first experiment, a single plane object USAF was recorded, and the raw hologram is shown in Figure 8a. The four phase-shifted holograms corresponding to phase-shiftes 0, π/4, π/2 and 3π/4 are shown in Figure 8b–e, respectively. The reconstruction result by numerical backpropagation of the complex hologram synthesized by combining the four phase-shifted holograms is shown in Figure 8f. The experiment was repeated for a two-plane object composed of a USAF and NBS object with a spacing of 65 mm. The single and phase-shifted holograms are shown in Figure 8g–k, respectively. The reconstruction results for USAF and NBS are shown in Figure 8l,m, respectively.

### 4.2. Conclusions

In conclusion, we have demonstrated FINCH using a GP lens as a compact imager with a low cost than all the versions of FINCH discussed in the previous sections. We believe that this approach will encourage the adaptation of FINCH to other imaging technologies and applications.

## 5. Fresnel Incoherent Correlation Holography with Non-Linear Reconstruction (Vijayakumar Anand and Saulius Juodkazis)

### 5.1. Status

**Figure 9:**
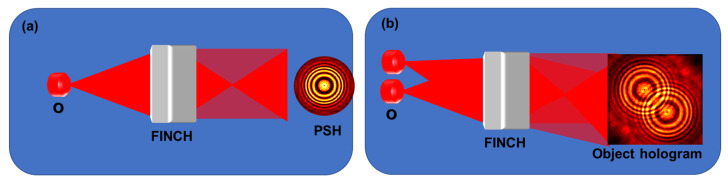
(**a**) Fresnel hologram obtained for a point and (**b**) Fresnel hologram obtained for two points.

In Section 2, Section 3, Section 4 and Section 5, novel optical architectures and optical components have been proposed to convert the required multiple temporal phase-shifted intensity patterns into spatially distributed spots. The above studies are notable advances as it prepared FINCH for recording dynamic scenes. Section 2 and Section 3 demonstrated spatial multiplexing approach, while Section 4 and [13] demonstrated polarization multiplexing approach. The spatial multiplexing approach results in a reduced field of view while the polarization multiplexing affects the signal to noise ratio. Taking the above effects into consideration, in 2020, FINCH was realized with pattern recognition [14]. For a single point, a FINCH system generates a Fresnel hologram, as shown in Figure 9a; and for two points, the FINCH system generates two such Fresnel holograms, which are summed as shown in Figure 9b. So, if the Fresnel hologram for a single point, i.e., the PSH is known, then it should be possible to reconstruct any object hologram by a cross-correlation as FINCH is a shift-invariant system.

Previous studies on cross-correlation revealed that correlations by a matched filter and phase-only filter were not optimal as they generate background noise and prevent the optical system from reaching the maximum resolution defined by the numerical aperture of the system [58,59]. In 2018, Rosen proposed a non-linear reconstruction method consisting of tuning the magnitude of the spectrum of PSH and object hologram (OH) until minimum entropy was obtained [60]. This approach toadied in reducing the background noise in imaging systems with reconstruction by cross-correlation and also enabled achieving the maximum lateral resolution of the imager. The pattern reconstruction problem was solved using the non-linear reconstruction method. The reconstruction is given as $I_{R}=\left|{ℱ}^{-1}\left\{{\left|{\tilde{I}}_{\mathrm{PSH}}\right|}^{\alpha }exp\left[i\;arg\left({\tilde{I}}_{\mathrm{PSH}}\right)\right]{\left|{\tilde{I}}_{\mathrm{OH}}\right|}^{\beta }exp\left[-i\;arg\left({\tilde{I}}_{\mathrm{OH}}\right)\right]\right\}\right|$ where α and β are tuned between −1 and 1, to obtain the minimum entropy given as $S\left(p,q\right)=-\sum^{\;}\sum^{\;}\phi \left(m,n\right)log\left[\phi \left(m,n\right)\right],$ where $\phi \left(m,n\right)=\left|C\left(m,n\right)\right|/\sum_{M}\sum_{N}\left|C\left(m,n\right)\right|$, (*m,n*) are the indexes of the correlation matrix, and *C*(*m,n*) is the correlation distribution.

**Figure 10:**
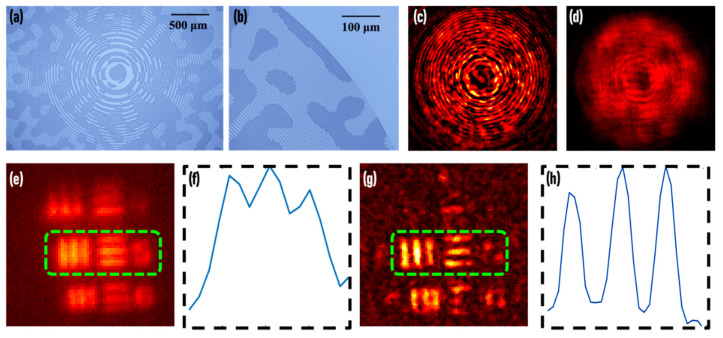
Optical microscope image of the (**a**) central part and (**b**) the outermost part of the fabricated diffractive lens. Images of the (**c**) PSH and (**d**) object hologram. (**e**) Direct imaging at one of the focal planes of the diffractive lens and (**f**) plot of averaged intensity values of the horizontal and vertical gratings shown in the green dotted box. (**g**) Reconstruction results and (**h**) plot of averaged intensity values of the horizontal and vertical gratings shown in the green dotted box. (Adapted from [14].)

Since, only a single shot is needed in this approach instead using an SLM, the beam modulator was fabricated as randomly multiplexed Fresnel lenses [1] using electron beam lithography. The optical microscope images of the diffractive element are shown in Figure 10a,b. The images of the PSH and object hologram for the USAF object Group 5, Elements 5 (50.8 lp/mm) and 6 (57 lp/mm) are shown in Figure 10c,d, respectively. The direct imaging result recorded at one of the focal planes of the diffractive element and the plot of the averaged intensity of the horizontal and vertical gratings are shown in Figure 10e,f, respectively. The reconstructed image (*α* = 0, *β* = 0.6) is shown in Figure 10g and the plot of the averaged intensity of the horizontal and vertical gratings is shown in Figure 10h. Comparing Figure 10f,h, an enhancement in visibility is seen in FINCH.

### 5.2. Future Challenges

The pattern recognition approach to FINCH has reduced the required number of camera shots from three to one. There are several drawbacks that are noted in this approach. It is necessary to record the PSH of the system using a pinhole and the resolution limit cannot exceed that of the pinhole. This section began with an argument that spatial multiplexing [33,34] and polarization multiplexing [35] reduced the field of view and the signal to noise ratio but this method also suffers from a low signal to noise ratio and the field of view appears lower than direct imaging, as shown in Figure 10e,g. Further studies are needed to understand this approach and achieve the theoretical limits of the field of view and the signal to noise ratio. Furthermore, the beam characteristics of the two interfering beams were not manifested in the imaging characteristics in this correlation approach. A recent simulative study revealed that the performance of FINCH in the pattern recognition approach was nearly independent of the characteristics of the interfering beams [36]. Therefore, the methods developed by Bouchal and Bouchal using vortex filter could not applied for edge enhancement [38] in this case. An iterative algorithm was designed recently to generate synthetic PSHs, which when correlated with the original PSH creates a donut-shaped intensity. This synthetic PSH, when correlated with object holograms, generated edge-enhanced image reconstructions. This direction needs to be explored further to understand the capabilities and limits of synthetic beam shaping [61].

### 5.3. Conclusions

The single-shot capability of FINCH can be achieved using two approaches. The first method is based on converting the temporal shots into spatially distributed shots by spatial and temporal multiplexing. The second approach uses pattern recognition. Another recent notable approach was the semi-analytical and logical approach developed by Tahara et al., reducing the number of camera shots to two [62]. This is a potential approach which needs to be explored further to determine the possibility of achieving a single-shot FINCH without sacrificing other qualities of FINCH such as super-resolution, the field of view and the signal to noise ratio.

## 6. FINCH Based on Metasurfaces (Hongqiang Zhou and Lingling Huang)

### 6.1. Status

**Figure 11:**
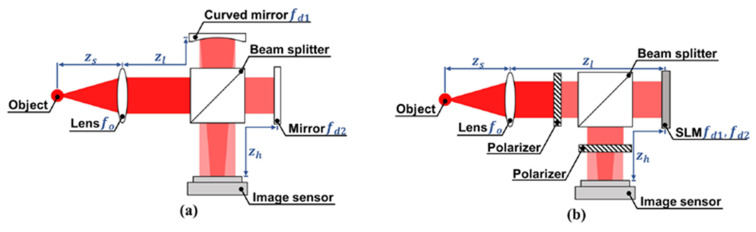
FINCH optical setup: (**a**) a Michelson interferometer-type imaging system; (**b**) a common-path interferometertype based on SLM imaging system. Reprinted with permission from [12]. Copyright (2021) Optical Society of America.

**Figure 12:**
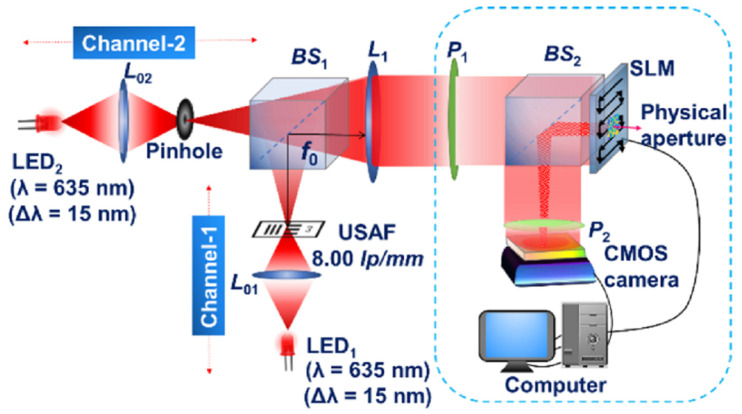
COACH optical setup using a synthetic aperture for super-resolution imaging. Reprinted with permission from [66]. Copyright (2021) Optical Society of America.

FINCH can be used to image 3D objects without phototoxicity to and bleaching of the living cells/organisms [7,17]. In a linear spatial-invariant imaging system, the theoretical lateral resolution of FINCH is twice as high as a conventional coherent imaging operation [40], which can be improved further using structural illumination [22]. The previous studies aim mainly at special applications or targets, such as biological cells or fluorescent samples. Experimental performances can be improved through optical configuration, such as by using a Michelson interferometer or a spatial light modulator (SLM) [12], as shown in Figure 11. Such an optical imaging system has tolerance to random noise, reconstruction distance, image magnification and slight aberrations [63,64]. However, multiple phase-shifting holograms are needed for twin-image-free reconstruction. The time needed and optical setup mismatch are the main limitations. In recent years, a new type of incoherent digital holograms, COACH, was developed, as shown in Figure 12. COACH uses a computer-generated random phase mask to replace the diffractive splitter lens mask loaded on the SLM in the FINCH technology. Therefore, the PSH of the system no longer has the form of a Fresnel zone plate but tends to have a certain spatial range. Through the cross-correlation operation between the PSH and the sample hologram, COACH realizes the three-dimensional reconstruction of sample information. It can reconstruct multidepth objects with a higher axial resolution than FINCH [29,59]. However, the FINCH and COACH imaging systems need stable and unmovable configuration for recording multiple holograms and high-quality reconstruction. Furthermore, the PSH needs to be pre-measured after building the system [60,65,66]. Hence, the minimization and integration of either FINCH or COACH without a complex optical system are highly desirable.

Metasurfaces have received great research interest in recent years due to their superior characteristics that are not available in naturally occurring materials [67,68,69,70]. Metasurfaces consist of a series of subwavelength ultra-thin nano-antennas arrays. Each meta-atom can modulate independently phase, amplitude or polarization and other fundamental properties of light. Usually the meta-atoms are subwavelength, which is significantly smaller than the pixels of commercial SLM. Therefore, metasurfaces can provide a larger field of view (FOV) and large space–bandwidth product (SBP). More importantly, metasurfaces can perform multiple functionalities through simultaneously tailoring several properties of light, such as polarization and phase manipulation simultaneously, and real space and ***k*** space manipulation simultaneously. Hence, various ultra-broadband or multiwavelength behaviors based on metasurfaces have been proposed and demonstrated through smart design and optimization. Metasurfaces have broad applications in metalens, holography, optical encryption, display, beam shaping, active modulation, etc. [71,72,73,74].

**Figure 13:**
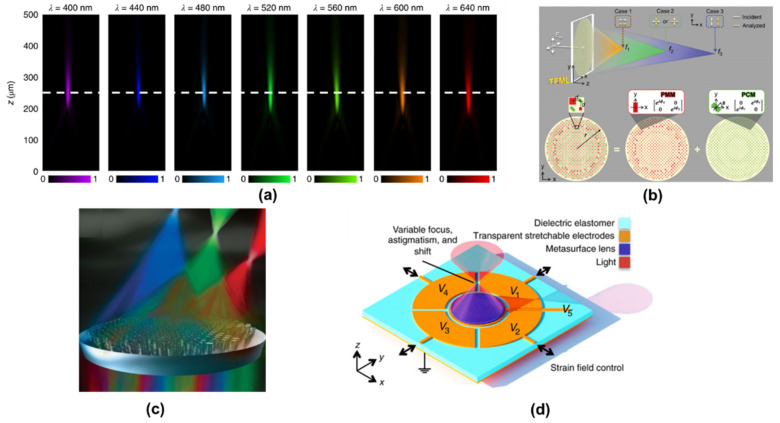
Multifunctional metalens applications. (**a**) Experimental results of achromatic metalenses. Reprinted with permission from Macmillan Publishers Ltd.: Nature Nanotechnology [72]. Copyright (2018). (**b**) Schematic illustration of the proposed twofold polarization-selective trifoci metalens (TFML), which establishes three foci at distinct focal planes depending on the linear polarization of incident and transmitted light. Reprinted with permission from Wiley Publisher: Advanced Optical Materials [78]. Copyright (2019). (**c**) Schematic of a metalens that simultaneously focuses and disperses the incident light. Reprinted with permission from Science [79]. Copyright (2018). (**d**) Schematic of the device in which a metalens and a DEA with five addressable electrodes are combined to allow for electrical control over the strain field of the metasurfac. Reprinted with permission from Science Advances [81]. Copyright (2018).

Metalens, an important branch of metasurfaces, have shown great capabilities for minimization of traditional optical imaging devices. Various types of metalens have been developed, including achromatic metalens, metalens for spectrum imaging, and bifocus or multifocus lens [72,75,76,77]. As shown in Figure 13, several typical examples are demonstrated. The achromatic metalens can perform the same focusing and imaging abilities for a broadband spectrum. While through polarization and phase modulation, metalens can achieve tri-foci focusing function according to selective polarization channels [78]. Smart metalens can also choose to achieve spectral focus imaging and disperse at multiple wavelengths [77,79]. Furthermore, through combining with Micro-Electro-Mechanical Systems (MEMS) or dielectric elastomer actuators (DEA), metalens can realize tunable focal lengths and dynamic beam steering [80,81]. These types of metalenses have great potential in incoherent holographic applications in the visible range because such metalenses can reduce the size of the FINCH imaging system and greatly reduce hardware costs.

### 6.2. Current and Future Challenges

As mentioned, FINCH is one potential imaging technology suitable for fluorescence three-dimensional imaging. The current mainstream FINCH systems are mostly based on diffractive lenses and reflective-type SLM configurations. Therefore, the huge optical system is a major limitation. It is often difficult to operate in some practical applications, such as on-chip devices. Particularly in micro-nano fluorescence holography, the huge optical path system needs to be compressed and integrated. The encoding and recording of multiple images also increase the time cost. This is not conducive to the recording of real-time holograms. While SLM has many advantages such as dynamic refresh, this technique is limited by its field of view.

### 6.3. Advances of Metalens to Meet Challenges of FINCH

**Figure 14:**
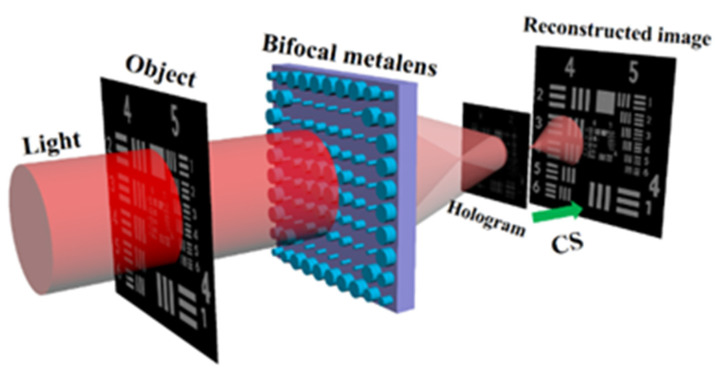
Schematic of coaxial holography and compress sensing reconstruction based on bifocal metalens [37]. Copyright (2020) Optical Society of America.

To solve the above challenges, a simple FINCH setup based on bifocal metalens has been proposed and demonstrated [37]. The metalens is an ultra-thin two-dimensional flat device composed of cylinder nanofins designed based on Huygens’ principle. Metalens can form two real images of an object along the optical axis, and form self-interference in the overlapping region, as shown in Figure 14. Afterwards, the real-time recorded digital hologram in the overlapping area is analyzed through compressive sensing algorithm, which can reconstruct the 2D or 3D object. This setting can ease the traditional FINCH system and is promising in replacing the combination of diffractive lens and SLM in FINCH. Because each meta-atom is isotropic, the bifocal metalens is polarization insensitive. Meanwhile, as a passive device, the bifocal metalens does not require energy consumption. It greatly reduces the size of the FINCH system. Therefore, it simplifies the recording process and improves application capability. The lightweight configuration provides a new way for the integrated and portable FINCH experimental system.

In future work, by optimizing metalens/metasurfaces, a wide-band, high-efficiency, large-size imaging function can be realized. The accurate focal length of the metalens has an important influence on the imaging of the FINCH system. The different response of the delicately designed meta-atoms to the wavelength may cause the change of its focal length, thereby affecting the imaging position. Therefore, optimal new type materials such as titanium dioxide TiO_2_ achromatic metalens can record and reconstruct incoherent fluorescence holography with higher imaging performance in the visible light or UV range.

### 6.4. Concluding Remarks

In summary, FINCH has important applications in the field of biomedical three-dimensional holographic imaging. There are already many methods based on FINCH to improve imaging performance and speed. However, in actual applications, an integrated, low-cost, portable device is required. The emergence of metalens and multifunctional metasurfaces can conquer these challenges due to their following advantages: they have flexible wavefront modulation and a compact footprint, and they are easy to fabricate and integrate. For future development of such techniques with small footprints and snapshot detection/reconstruction, achromatic behavior and dynamic modulation metasurfaces are required, because compared with the SLM-based FINCH system, the processed and packaged bifocal metalens are immutable. Nevertheless, by integrating with phase-shifting materials, liquid crystal or other active materials, such tunable metalens can be adapted to various application scenarios such as biological detection, optical coherent tomography, and virtual and augmented reality. The achromatic performance can ensure incoherent light illumination, which can surpass the narrow bandwidth, and can provide more vivid images with spectral information.

## 7. Vortex FINCH in Spiral and Localization Microscopy (Petr Bouchal and Zdeněk Bouchal)

### 7.1. Status

Optical vortices have helical wavefronts with screw phase dislocations causing the spinning of electromagnetic energy and transferring the orbital angular momentum. The vortex fields significantly influenced the development of optical manipulations and communications and gave rise to new imaging microscopy techniques. Here, we present vortex FINCH modalities, which will allow us to take full advantage of incoherent holographic imaging in spiral and localization microscopy. We demonstrate spiral FINCH imaging using the vortex impulse response function and providing the controllable edge-contrast enhancement in a three-dimensional (3D) digitally reconstructed image. In another approach, we implement the double-helix point spread function into FINCH imaging to spatially localize point-like emitters by gaining depth from diffracted rotation. We discuss spiral and localization imaging for both optical designs using natural optical vortices and digital implementation. In the digital alternative, we introduce vortex fields when reconstructing correlation records acquired in the standard FINCH arrangement.

FINCH technology was established as a well-proven approach to incoherent digital holography allowing imaging of three-dimensional (3D) amplitude objects in spatially incoherent light. Following the discovery of the method almost 15 years ago [1,17,24], the research resulted in revealing unique FINCH imaging capabilities [8,30,31], designing optimized arrangements [27,82,83], and utilizing new optical elements provided by advanced technologies [35,84]. FINCH has succeeded as an efficient and easy-to-implement imaging tool of fluorescence microscopy, capable of overcoming the diffraction resolution limit [85]. Simultaneously with FINCH developing, specific light fields represented by vortex and diffraction-free beams brought progress into photo-activated localization imaging [86] and gave rise to spiral microscopy [87,88]. The effects used were mostly associated with the phase topology of optical vortices; hence, interference conditions were necessary.

Here, we present concepts combining the imaging potential of coherent optical vortices with the advantages of FINCH experiments conducted using incoherent light. Our efforts resulted in optical and digital vortex FINCH modalities, which we demonstrate in their use for isotropic and anisotropic edge-contrast enhancement of 3D holographic images [38] and for super-localization of point-like emitters [89].

**Figure 15:**
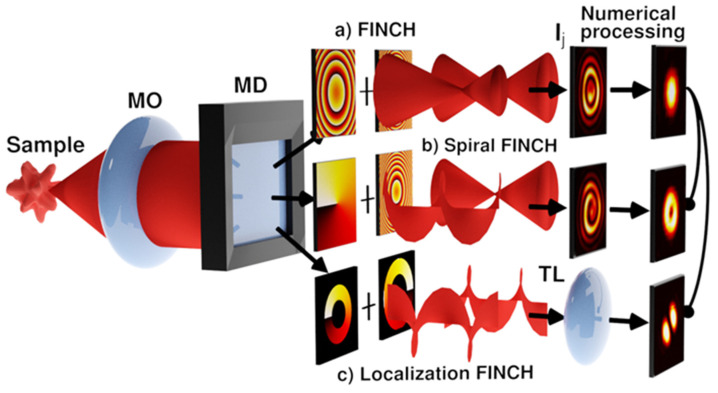
Illustration of vortex FINCH and spatial light shaping in (**a**) standard, (**b**) spiral, and (**c**) localization FINCH imaging. MO—microscope objective; MD—modulation device; TL—tube lens.

Our research is focused on introducing a vortex impulse response into FINCH, which will allow us to take advantage of this method in spiral and localization imaging. In FINCH, the holograms arise as an incoherent superposition of point self-interference patterns. By reconstructing them numerically, the impulse responses are obtained, which are processed coherently to obtain a 3D image of the sample. This mixed coherence regime is suitable for engaging vortex fields into FINCH operation. In Figure 15, the standard FINCH (Figure 15a) is compared to vortex modalities for the spiral and localization imaging. The edge-contrast enhancement in the spiral imaging is achieved using a vortex impulse response. Its amplitude represents a ring, while the phase changes linearly with the azimuthal coordinate φ as exp(imφ), where m is the topological charge. In the optical embodiment, a helical reference wave is used creating a spirally shaped point correlation pattern that provides the vortex impulse response after numerical reconstruction (Figure 15b). In the digital vortex FINCH, the vortex impulse response is created by spiral phase modulation applied to the numerical processing of standard FINCH records. In 3D localization experiments, the depth is gained from the angular rotation of the double-helix point spread function (DH PSF) created by the interference of vortex beams with the opposite topological charges (Figure 15c).

#### 7.1.1. Demonstration of Spiral FINCH Imaging

**Figure 16:**
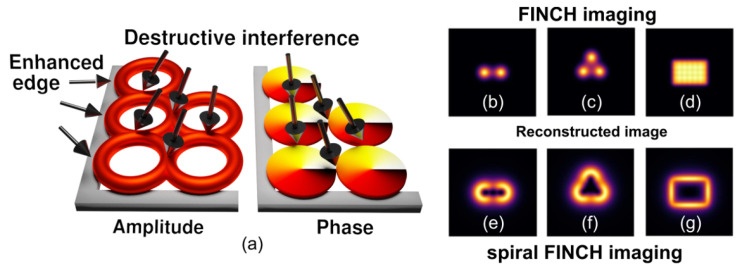
Destructive interference of vortex impulse responses (**a**), and demonstration of intensity images created for (**b**) and (**e**) two-point object, (**c**,**f**) three-point object, and (**d**,**g**) 7 × 5 array of point objects.

**Figure 17:**
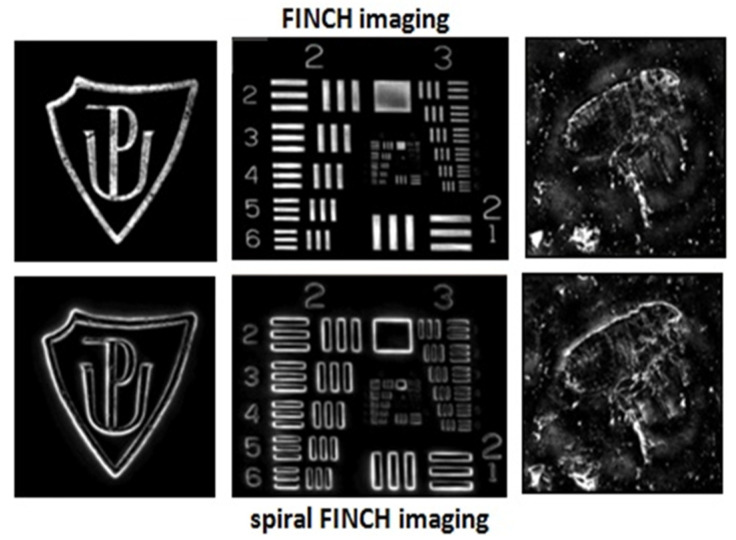
Imaging of the Palacký University sign, USAF test, and flea created using FINCH and spiral FINCH under incoherent illumination.

The image formation in spiral FINCH is demonstrated in Figure 16. A spiral correlation pattern is created for a point object using a vortex reference wave generated by a phase modulation device. An annular impulse response with a vortex phase is reconstructed from three phase-shifted spiral correlation patterns (Figure 15b). The spiral patterns arise simultaneously for all points of a 3D object, and their intensities are superimposed under incoherent illumination. The image is reconstructed coherently; hence, the image intensity is affected by the destructive interference stemming from the vortex phase (Figure 16a). This effect became the basis of spiral microscopy using coherent light [87,88]. When two point sources of the same amplitude are imaged, the resulting intensity disappears just between the image centers due to the vortex interference. The images of the two-point object restored by FINCH and spiral FINCH are compared in Figure 16b,e. Between the bright spots created by FINCH for the three-point object (Figure 16c), a dark area appears in spiral FINCH because the vortex images interfere destructively there (Figure 16f). In the object area where the amplitude of the emitted or reflected light remains homogeneous, the intensity vanishes when spiral FINCH restores the image. This effect is demonstrated by the imaging of a rectangular area formed by 7 × 5 array of point sources of the same amplitude (Figure 16d,g). The vortex interference provides the edge-contrast enhancement in which the intensity is significant only in the regions of amplitude gradients. The spiral edge-contrast enhancement is well documented by imaging the Palacký University sign, USAF test, or flea (Figure 17). In the digital alternative, the vortex impulse response is introduced by the spiral phase modulation performed numerically when processing records acquired without a vortex reference wave [38].

#### 7.1.2. Demonstration of Localization FINCH Imaging

**Figure 18:**
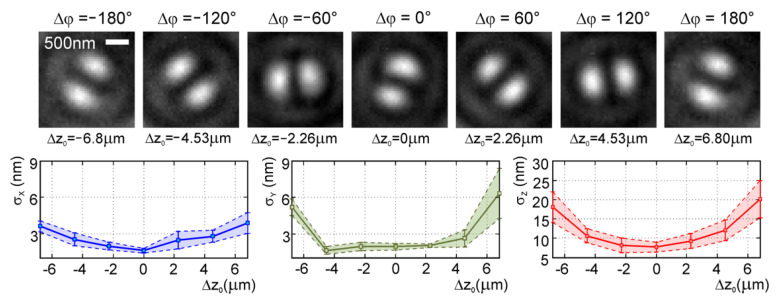
Localization of 100 nm gold beads by the vortex FINCH microscope: DH PSF and standard deviations for x, y, z coordinates evaluated at seven different depths in the axial range of 13.6 μm.

The proposed localization imaging utilizes FINCH capability of splitting light waves from individual object points into two new waves interfering even under incoherent illumination. This is achieved by amplitude and phase modulation in the back focal plane of the microscope objective. The incident light is transmitted through two rings in which the phase of light changes with the azimuthal angle φ as exp(iφ) and exp(−iφ), respectively (Figure 15c). By performing the optical Fourier transform by a tube lens, two non-diffracting vortex beams of opposite topological charges are generated for each point source in the object space. Interference of non-diffracting vortex beams creates a two-lobe diffraction image spot known as the double-helix point spread function (DH PSF) [90]. When the point source generating the pair of optical vortices moves axially, the light phase in the rings alters, causing that the DH PSF rotates angularly [91]. By determining the lateral position of the DH PSF and the angular rotation of its lobes, the point object is accurately localized in 3D space. A flexible non-diffractive vortex microscope was designed for 3D depth-enhanced super-localization of dielectric, metal, and fluorescent nanoparticles by implementing the DH PSF into FINCH imaging [89]. Subwavelength gold and polystyrene beads were localized with isotropic precision below 10 nm in the axial range of 3.5 μm and the axial precision reduced to 30 nm in the extended range of 13.6 μm (Figure 18). Applicability of the method was tested by fluorescence imaging of LW13K2 cells and localization of cellular proteins [89].

In the digital embodiment of the localization FINCH imaging, vortex interference is no longer used, and instead, a single non-diffracting vortex beam is generated to obtain depth information. The point objects to be localized are recorded in a standard FINCH experiment, and their images reconstructed using the Fresnel transform. After the numerical Fourier transform, the spatial spectrum of the image is filtered to pass a single radial frequency. At the same time, the spiral phase modulation with the unitary topological charge is applied. After the inverse Fourier transform, a non-diffracting vortex image spot is created, whose complex amplitude encodes the depth information. The amplitude A(r) of the image spot is annular, while the phase alters as exp(iφ+iΔΦ), where r and φ are the cylindrical coordinates in the image plane. The additional phase shift ΔΦ is proportional to the depth being measured, ΔΦ=βΔz, where β is the proportionality constant depending on the parameters of the experiment, and Δz is the position of the point object relative to the focal plane of the microscope objective. The real part of the complex amplitude A(r)cos(φ+βΔz) creates two lobes on the ring, which rotate when the axial position Δz of the point object varies. With this technique, accurate 3D localization is possible by processing a single record, making the localization imaging capable of tracking dynamic processes [92].

### 7.2. Conclusions

This paper demonstrates that the coherent effects associated with the phase topology of optical vortices can be effectively transferred to FINCH imaging realized with incoherent light. The spiral and localization imaging techniques are examined as perspective applications of the presented vortex FINCH modality. The connection of FINCH imaging with a geometric-phase transformation providing spin-to-orbital angular momentum conversion is another important topic for further research. In this way, a 3D orientation imaging of dipolar nanoobjects can be realized.

## 8. Three-Dimensional Reconstruction for Living Cell by Using a Femtosecond Laser-Based Phase-Shifting Fresnel Incoherent Digital Hologram (Bang Le Thanh, Munkh-Uchral Erdenebat, and Nam Kim)

### 8.1. Status

FINCH was proposed as a correlation between the object and Fresnel zone plates (FZP). It is based on a single on-axis interferometer and does not require any movement or multiplexing image of the scene [36,39]. So, the FINCH system is widely applied in microscope and imaging methods with a synthetic aperture.

Otherwise, a phase-contrast microscope is very effective for observing the specimens, examining cells in a natural state, and studying biological processes. Observing a living organism in its natural environment can provide more information on specimens that need to be killed, fixed, or stain to view under the microscope. The three-dimensional (3D) reconstruction for living cells in the phase-contrast microscope is provided with high-contrast [93,94,95] and high-resolution images [95]. In addition, advances in the phase-contrast microscope, especially those that incorporate technology, enable the presence of the internal structure of samples [96] and can even detect a small number of protein molecules [97]. However, the cell is a much small object, and its composition includes protein and water; so, if the intensity of illumination is very weak, the focal points are often superposed by light out of focus. Consequently, capturing the living cells, especially in the 3D reconstruction, is very difficult to acquire the fine-resolution images with the conventional method. To overcome this problem, some existing methods use a confocal laser scanning fluorescence microscope with excitation radiation of He-Ne lasers [98], argon/krypton ion lasers, He-Cd lasers, and laser diodes or multiphoton excitation method of distinct object planes. Or using the holography method for focus plane detection [99]. However, these methods cannot achieve the progress of scanning fluorescence microscope both depth information and resolution image.

When the FINCH is applied to the holographic 3D display, it can be an effective way to reconstruct 3D images for living cells where a femtosecond laser is utilized as a laser illumination [100]. The feature of a femtosecond laser is a high level of light intensity, the extreme stability of the optical setup, and the supply of narrow bandwidth light. In addition, when a specimen with a much thicker size is used, the quality of reconstructed im-ages is often surrounded by bright areas, and the accuracy of the reconstructed image is decreased. This problem can be solved by the light emitted from living cells that are dyed by fluorescence after cells are illuminated by a femtosecond laser with 300–600 nm wavelength. Thus, each point emission might be inside a cell or on the surface of a cell is similarly one object point. By using a spatial light modulator (SLM) and CCD camera for the FINCH system, a 3D visualization of the cell can be captured with full depth information [101].

In this report, we introduce a method for obtaining phase-shifting with higher phase contrast in the reconstructed 3D hologram by using an SLM and femtosecond laser for living cells. By changing the phase fringe, a proposed method can record phase-shifting in reconstructed specimens with different thicknesses instead of depending on a fix-phase of phase plate and phase annulus in a conventional phase-contrast microscope. Therefore, the proposed system can achieve the flexibility to choose the phase and to calculate the phase on the annulus plane in accordance with the thickness of each object to observe the object more precisely.

**Figure 19:**
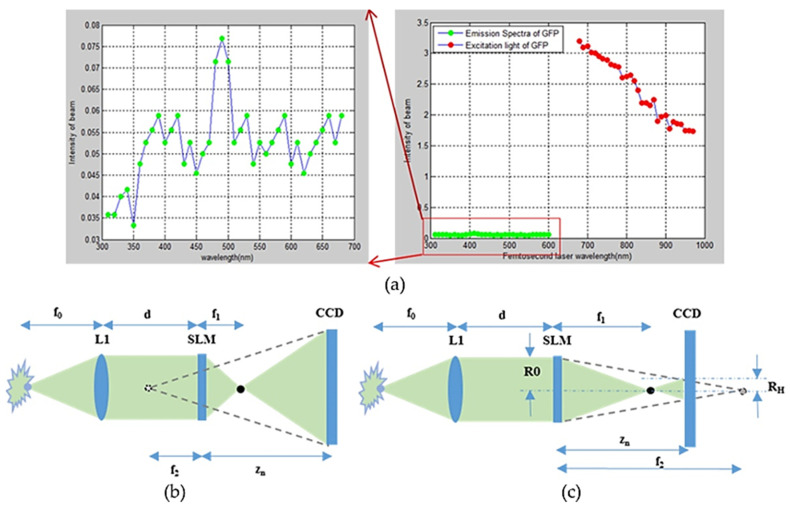
(**a**) Excitation and emission spectra of the living cell, FINCH with two diffractive lenses: (**b**) one is positive, and the other is negative, and (**c**) both are positive (adapted from [39]).

Figure 19a describes the relationship between the wavelengths of the excitation light and the light emitted from the cell. The wavelength of the emitted light of a femtosecond laser is changed as 680–1024 and 310–660 nm, and the emitted light with 500 nm wavelength is the highest intensity.

The FINCH described in Figure 19b is positive and the other is negative with SLM position. A 3D object has many source points and hence we obtain a hologram of the whole object. The diffractive object element on SLM can be expressed as a function:

(1) $$R\left(x_{D},y_{D}\right)=\frac{1}{2}+\frac{Q}{2}\left(-\frac{1}{f_{2}}\right)\exp\left(i\theta \right)$$

where θ is the phase distribution of the reflection masks displayed on the SLM, and *Q* function is given by:

(2) $$Q\left(b\right)=\exp\left[i\pi b\left(\frac{x^{2}+y^{2}}{\lambda }\right)\right]$$

where *λ* is the central depth plane. The hologram of an object point located at (0, 0, *z_s_*):

(3) $$I_{P}=A|Q\left[{\left(\frac{f_{0}\left(f_{0}-z\right)}{z}+d+z_{n}\right)}^{-1}\right]+{Q\left[{\left(\frac{f_{2}f_{0}\left(f_{0}-z\right)+f_{2}zd_{1}}{zf_{2}-f_{0}\left(f_{0}-z\right)-zd_{1}}+d_{2}\right)}^{-1}\right]\exp\left(i\theta \right)|}^{2}$$

To remove the twin image and the bias term, three holograms are recorded with a different phase constant *θ*. The final hologram is described as:

(4) $$\begin{array}{l} U\left(x,y\right)=U_{1}\left(x,y\right)\left[\exp\left(-i\theta_{3}\right)-\exp\left(-i\theta_{2}\right)\right]+U_{2}\left(x,y\right)\left[\exp\left(-i\theta_{1}\right)-\exp\left(-i\theta_{3}\right)\right]+\\ U_{3}\left(x,y\right)\left[\exp\left(-i\theta_{2}\right)-\exp\left(-i\theta_{1}\right)\right] \end{array}$$

Additionally, the reconstructed object is given by:

(5) $$O\left(x,y,z\right)=U\left(x,y\right)\times \exp\left[\frac{i\pi }{\lambda z}\left(x^{2}+y^{2}\right)\right].$$

The overlapping object points on two adjacent layers are identified as:

(6) $$Max\left\{Reconstruction\left(z_{1},z_{2}\right)\right\}\approx Max\left\{Reconstruction\left(z_{3},z_{2}\right)\right\}$$

where *z*_1_ < *z*_2_ < *z*_3_. *z*_1_ and *z*_3_ are the same as the distance of the recorded hologram.

**Figure 20:**
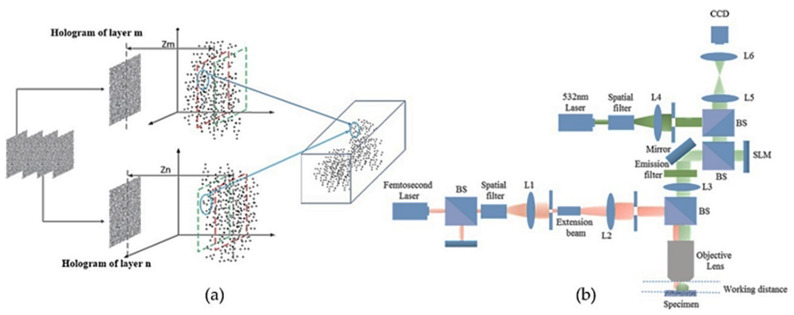
(**a**) Overlapping object points after reconstruction of each layer in the hologram, and (**b**) the optical setup for the proposed system (adapted from [39]).

**Figure 21:**
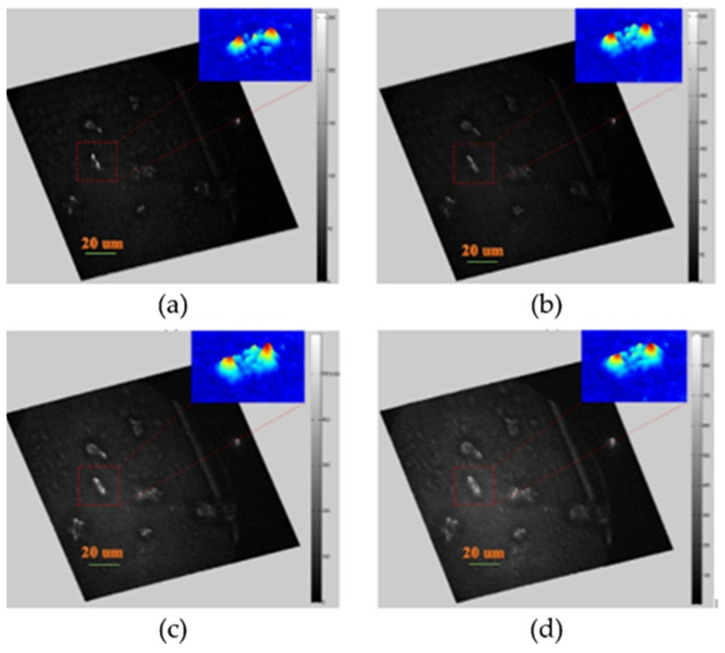
Reconstructed images for cell with (**a**) 2 layer, (**b**) 4 layer, (**c**) 6 layer, and (**d**) 8 layer holograms (adapted from [39]).

**Figure 22:**
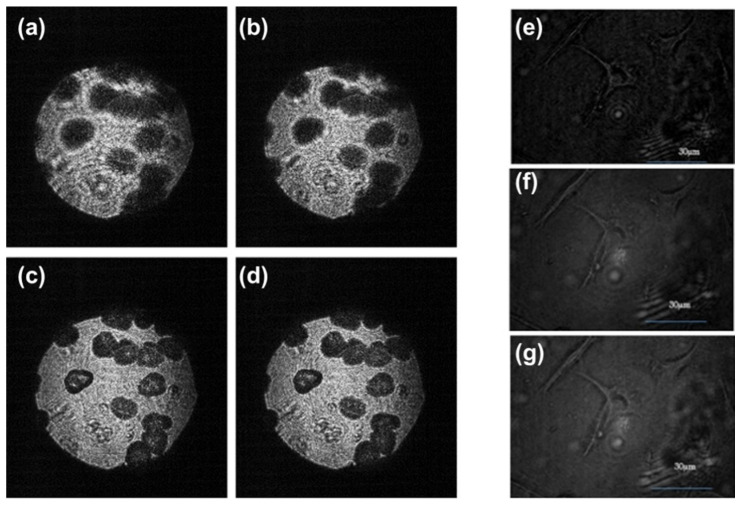
Recorded images with a (**a**) 2 step phase fringe = 0, π/2 (left-top); (**b**) 4 step phase fringe = 0, π/2, π/4, 7π/4 (right-top); (**c**) 6 step phase fringe = π/4, 7π/4, 3π/8, 13π/8, π/8, 15π/8, 0, π/2 (left-bottom); and (**d**) 8 step fringe with phase = 3π/8, 13π/8, π/8, 15π/8, 0, π/2, π/4, 7π/4 (right-bottom). Reconstructed images of the living cell B16F10 with a (**e**) 2 step phase fringe (top), (**f**) 6 step phase fringe (middle) and (**g**) 8 step phase fringe (bottom).

The overlapping object points at different layers are shown in Figure 20a and the optical configuration of this study is shown in Figure 20b. The reconstruction results of cells for 2 layers, 4 layers, 6 layers and 8 layers are shown in Figure 21a–d, respectively. The recorded images: 2 steps with phase = 0, π/2; 4 steps with phase = 0, π/2, π/4, 7π/4; 6 steps with phase = π/4, 7π/4, 3π/8, 13π/8, π/8, 15π/8, 0, π/2; and 8 steps with phase = 3π/8, 13π/8, π/8, 15π/8, 0, π/2, π/4, 7π/4 are shown in Figure 22a–d, respectively. The reconstructed images of the living cell B16F10 with a 2 step phase fringe, a 6 step phase fringe and an 8 step phase fringe are shown in Figure 22e–g, respectively.

### 8.2. Conclusions

In this report, we summarized recent progress on overcoming the limitation of conventional FINCH display. It may seem to contradict the common belief that only well-designed optical components can be used for imaging 3D object enhancement. However, multiple scattering can offer new possibilities in small objects, especially living cells with illuminated light that is very weak. With the application of a femtosecond laser and an optical component with the FINCH system, the 3D visualization of the living cell including an inner structure was successfully reconstructed, whereas it was very difficult with the traditional method.

## 9. Single-Molecule Localization with FINCH (Peter Kner and Abhijit Marar)

### 9.1. Status

Fluorescence microscopy is a natural application of FINCH, and FINCH has been demonstrated on fluorescently labeled biological samples [1,17]. In particular, three-dimensional fluorescence imaging of cells should benefit because with FINCH, the entire three-dimensional volume can be imaged with only three camera exposures per color. Because FINCH imaging does not obey the Lagrange invariant, FINCH can achieve higher resolution than widefield fluorescence imaging [30,85]. Imaging with a lateral resolution of 132 nm has been demonstrated [16]. The axial resolution measured was 470 nm, similar to that of widefield imaging.

To improve the resolution beyond this, different super-resolution approaches must be considered. SMLM is a method for achieving super-resolution by imaging single emitters stochastically [102]. As each emitter is imaged, the emission is localized by determining the center of the imaged spot of light–because the emitters are nanoscale molecules, the spot is the system PSF. The precision with which the center of the PSF can be localized is determined by the noise in the imaging system. The brighter the spot, the more accurately the center can be determined, and the accuracy of SMLM is $\sim s/\sqrt{N}$ where *s* is the PSF width and *N* is the number of photons emitted. Although this accuracy is degraded by background. Single fluorophores emit a few hundred to a few thousand photons, and the accuracy of localization is typically 10 nm to 40 nm.

Localization of the widefield PSF is a two-dimensional imaging technique. The PSF is imaged onto the camera in two-dimensions, and the conventional PSF does not provide high-resolution information about the axial center of emission. SMLM can be extended to three-dimensions by modifying the PSF. The most common modification is to insert a weak cylindrical lens into the imaging path [103]. This creates an asymmetry in the PSF which changes orientation from above to below focus. This approach provides an axial resolution of ~70 nm over an axial range of ~1 µm. To increase the axial range and precision, novel PSFs have been engineered including the double-helix PSF and the tetrapod PSF [104,105]. Using the double-helix PSF, a lateral localization precision of 12 nm and an axial precision of 19 nm have been measured from ~9000 photons over an axial range of a bit less than 2 µm. The tetrapod PSF is designed to achieve a minimum Cramer-Rao Lower Bound over a specified axial range with a given background level. Measuring fluorescent beads, a localization precision of 15 nm in *x*, 12 nm in *y*, and 29 nm in *z* has been demonstrated over a 7 µm axial range from 6000 photons with 40 background photons per pixel. A disadvantage of the tetrapod PSF is that it is significantly larger than the conventional PSF. Therefore, the occurrence of overlapping PSFs is more likely, leading to decreased localization accuracy and precision [106].

Another approach to three-dimensional SMLM is based on quadri-wave shearing interferometry (QWSI) [107]. In this approach, multiple copies of the PSF are created near the image plane using a grating and are interfered at the camera. The image on the camera is then a conventional PSF with an interference pattern superimposed. The interference pattern encodes the axial position. Bon et al. used this approach, termed Self-Interference (SELFI), for SMLM to image 3D networks of actin filaments [108]. They demonstrated that the technique works well deep inside tissue, but the axial range is less than 2 µm. In Liebel et al. [109], the same approach, now called holographic fluorescence imaging, was used to track single particles over an 8 µm axial range. Beyond 8 µm, they saw deviations in the axial localization. They also used very bright emitters and did not perform SMLM imaging. The difference in axial range between the two approaches suggests that QWSI loses signal-to-noise ratio (SNR) away from focus as the PSF expands. A further difficulty for SMLM is that the emitters will be more likely to overlap away from focus. An advantage of FINCH is that the hologram image stays roughly the same size over the axial range.

**Figure 23:**
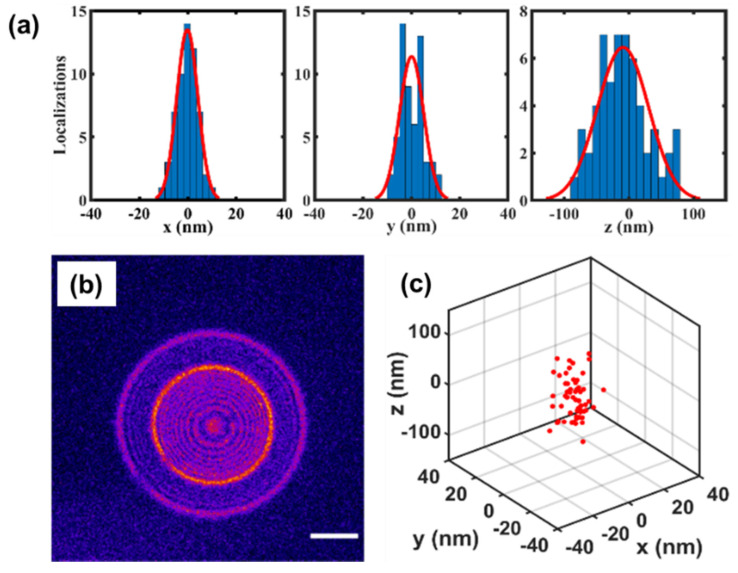
3D localization of a single 0.1 µm fluorescent bead. (**a**) Histograms of 68 localizations in *x*, *y*, and *z* of one single 0.1 µm red (580/605) fluorescent bead on a coverslip. The standard deviations of the measurements are $\sigma_{x}=\sigma_{y}=5\;\mathrm{nm}$, and $\sigma_{z}=40\;\mathrm{nm}$. (**b**) Representative hologram of a single bead acquired in one 50 ms exposure. Scale bar is 50 µm. (**c**) Scatter plot of localizations. Reprinted with permission from [40].

Applying FINCH to three-dimensional localization and SMLM should offer many advantages. Because FINCH has large axial range and an analytical reconstruction process, it avoids some of the difficulties with engineered PSFs such as the tetrapod. With FINCH, the PSH is convolved with a Fresnel propagator to create a two-dimensional image with a PSF that is narrower than the conventional PSF. Therefore, overlapping PSHs do not lead to a difficulty in the localization process. We have demonstrated localization with FINCH using fluorescent beads, Figure 23 [40]. With ~50,000 photons we were able to localize a particle with a precision of 5 nm laterally and 40nm axially, and we were able to reconstruct the PSF from as few as 13,000 photons. We were also able to demonstrate that strongly overlapping holograms can be reconstructed to form well-separated PSFs.

Single fluorophores typically produce a few hundred to a few thousand photons before switching off or photobleaching. The sensitivity of FINCH needs to be increased to allow for reliable localization in this regime. In the case of no background and pure Poisson noise, the large size of the hologram presents no difficulties. The reconstruction process adds no additional noise, and the reconstructed PSF will have high SNR. However, the hologram experiences more background than the traditional PSF since the hologram is larger, and so the precision of localization degrades in the presence of background. By calculating the Cramer-Rao Lower Bound for the FINCH PSH, we have shown that FINCH can achieve axial localization precision better than the astigmatic PSF and the Cropped Oblique Secondary Astigmatism (COSA) PSF with no background [110]. However, with background levels typically seen in fluorescence microscopy of single cells, the localization precision of FINCH degrades to ~40 nm for emission of 6000 photons–worse than the precision of the astigmatic or COSA PSF.

### 9.2. Future Work

The combination of FINCH and SMLM should make it possible to image whole cells with approximately 20 nm resolution without any sample movement. The ability to localize fluorophores throughout the entire thickness of the cell with FINCH and without any mechanical movement could substantially speed up 3D SMLM and remove any loss of precision due to mechanical movement. However, to achieve this goal, FINCH must be optimized for SMLM applications to increase the SNR for low signal levels. It must be optimized to detect a hologram of a few hundred to a few thousand photons in the presence of background levels on the order of 10 photons per pixel which is typical of biological SMLM. To achieve the highest precisions the light efficiency of the FINCH system must be maximized and the background must be as low as possible.

We feel this can be achieved by optimizing the Cramer-Rao Lower Bound on the precision which has been used to optimize the localization of the conventional PSF and design engineered PSFs such as the tetrapod [106,111]. Optimization should be performed with the imaging conditions in mind. The expected number of photons from the fluorophores and the expected background levels must be included in the process. Additionally, in realistic imaging conditions and given the larger size of the PSH compared to the PSF, it is likely that multiple holograms will overlap. Multiple emitters can also be included in the optimization process [112]. Current algorithms for axial localization with single-molecule FINCH do not achieve the Cramer-Rao Lower Bound, and new algorithms must be developed [110].

Other improvements to FINCH will also improve the outlook for SMLM. FINCH requires three phase-shifted images to separate the PSH from the background and the twin image. To optimally image stochastic emitters, it is better to acquire the PSH in a single shot. Some different approaches to single-shot imaging have been proposed which can be explored. Optical sectioning could also improve the performance by removing background from the reconstructed sections. Localization can be further improved by accounting for optical aberrations [11]. In SMLM, aberrations distort the PSF reducing the accuracy of localization. In FINCH, the aberrations can be calculated and corrected from the hologram. By correcting the aberrations, the localization will be improved. Adaptive Optics has been demonstrated with FINCH using a fluorescent plate imaged through a USAF target [113]. Correcting the hologram of weak emitters will be more challenging but will also allow each emitter to be corrected individually, resulting in an elegant solution to correcting field-dependent aberrations.

Finally, FINCH has led to many novel variations including COACH, Interferenceless-COACH (I-COACH), and hybrid FINCH-COACH [7]. In COACH, a random phase screen is used rather than producing a focus with a parabolic phase screen or lens. A series of PSHs is then taken over the imaging volume to calibrate the system. COACH has higher axial resolution than FINCH. In I-COACH, the interference is dispensed with so that I-COACH is a single-shot technique. So far, work on these techniques has consisted largely of demonstrations with artificial samples (as opposed to fluorescently stained biological samples). Applying novel FINCH approaches to SMLM will be an exciting avenue of research but light-efficiency will be a critical consideration for these approaches.

### 9.3. Conclusions

SMLM is already an established tool in biological imaging and has provided insights into many sub-diffraction structures including chromosome organization and the structure of the axon. FINCH has many potential advantages for 3D SMLM. FINCH can localize molecules over a 20 µm axial range. The holograms are reconstructed into 2D images with an analytical reconstruction process mitigating difficulties with overlapping emitters that many PSF engineering approaches face. In the background free case, the theoretical precision limit of FINCH compares favorably to other localization approaches.

Because the PSH is fairly large on the sensor, imaging weak fluorophore signals in the presence of background is challenging. Future work is needed to optimize FINCH for localization in the presence of background. As work continues on SMLM using FINCH, we are confident that performance will steadily improve and that many exciting improvements will be demonstrated including aberration correction of single emitters with FINCH and the application of different holographic methods to SMLM.

## 10. Incoherent Holography Lattice Light-Sheet (IHLLS) (Mariana Potcoava, Christopher Mann, Simon Alford and Jonathan Art)

### 10.1. Status

Biological tissue is complex in 3D. For example, neurons possess dendritic inputs and axonal outputs that show varied and convoluted 3D structures with rapid signaling at inputs and outputs distributed throughout these 3D spaces. Conventional imaging approaches do not allow for the millisecond data acquisition resolved in 3D space necessary to understand function. Confocal imaging, multiphoton imaging, and light sheet methods [114,115] all allow resolution of 3D space, but place limitations on speed and resolution necessary to reconstruct information by scanning points or 2D image planes. Furthermore, to reconstruct 3-dimensional structures the objective lens must be moved. This takes time, and at high-speed used in light sheet imaging, it causes mechanical distortion. We have developed holographic approaches that allow faster 3D image acquisition and obviate the need for movement of the objective lens.

**Figure 24:**
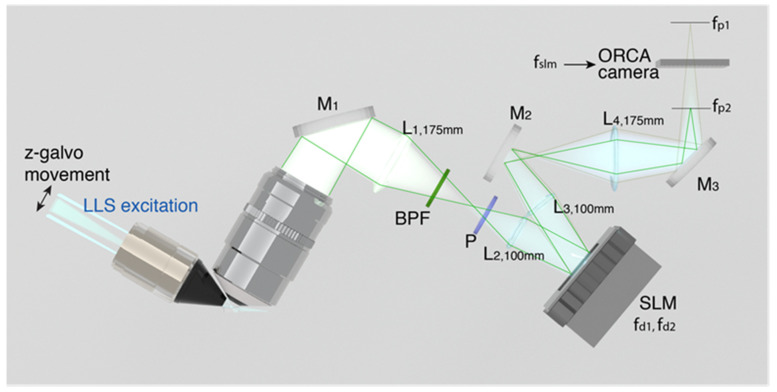
Schematics of the IHLLS systems with one diffractive lens of focal length, f_SLM_ = 400 mm (IHLLS 1L) and two diffractive lenses with focal lengths f_d1_ = 220 mm and f_d2_ = 2356 mm (IHLLS 2L). A collimated 30 Bessel beam is focused by an excitation objective lens which generates a lattice light sheet. It excites fluorophores in the focal plane and in/off the focal plane of the detection objective lens, which is a water immersed microscope objective MO (Nikon 25X, NA 1.1, WD 2 mm). The detection system also includes two pairs of lenses for beam size adjustment to fit the size of the SLM active area, L_1_ = L_4_ with focal lengths 175 mm, L_2_ = L_3_ with focal lengths 100 mm; mirrors M_1_, M_2_, M_3_; polarizer P; 40 nm band pass filter BPF centered on the 520 nm wavelength, spatial light modulator SLM, and CMOS camera. While the z-galvo and z-piezo are moved along the *z* axis to acquire stacks in LLS and IHLLS 1L**,** in IHLLS 2L only the z-galvo is moved at various z positions (Visualization 1 [41]). The diffraction mask in the excitation path was positioned for all experiments on the anulus of 0.55 outer NA and 0.48 inner NA. The detection magnification $M_{T-LLS}$ = 62.5 and the illumination wavelength $\lambda_{illumination}$ = 488 nm. The width of the light sheet in the center of the FOV is about 400 nm.

Our imaging technique [41], incoherent holography lattice light-sheet (IHLLS), Figure 24, uses the excitation technology of a lattice light-sheet (LLS) system [116,117]. However, it allows us to construct the 3D amplitude volume of complex objects such as neurons with resolutions comparable or better than the conventional LLS’s resolution in the diffraction limited dithering mode and can achieve an extended field of view (FOV).

In LLS, the excitation light is maintained within a plane caused by constructive interference of a lattice of self-reinforcing and intersecting Bessel beams that project through the sample. Images are obtained by viewing fluorescence orthogonally to the projection of the beam. In our approach, the imaging detection uses the FINCH principle [1,10,17,32,83]. FINCH uses self-interference of the emitted fluorescent light to create Fresnel holograms of a 3D object in combination with the phase-shifting concept from which a single channel on-axis interferometer creates three to four interference patterns but in which the interferometer’s beam splitter is replaced by an SLM. In this arrangement, each spherical beam propagating from the points of each 3D object is split into two spherical beams with different radii of curvatures. The interference patterns are added incoherently, to further create Fresnel holograms. These holograms are numerically processed by in-house diffraction software.

Implementation of IHLLS was addressed in two stages:

Build a copy of the LLS detection arm but using incoherent light (IHLLS-1L). The optical design optimization is performed using Opticstudio. This enabled us to compute the focal length ($f_{SLM}=400\;\mathrm{mm}$) of the single diffractive lens that was uploaded on the SLM, to match pixel sizes to a specific resolution in both conventional LLS and in IHLLS 1L. Similarly, the correct distances were computed between each optical component.

Upload onto the SLM two diffractive lenses super-imposed but with different focal lengths. These lenses are composed of randomly selected pixels (IHLLS 2L), to give access to various depths in the sample through which only the z-galvo was moved within the $\Delta z_{galvo}\;$displacement range. This range is centered at the objective reference focus position (the center of the camera FOV in the z-axis). Again, using Opticstudio, we designed a re-configurable optical system such that the two spherical beam displacement was equal at the camera plane to overlap precisely. After performing the optimization, we obtained the two focal distances of the two lenses as: $f_{d1}=220\;\mathrm{mm}$ and $f_{d2}=2356\;\mathrm{mm}$.

**Figure 25:**
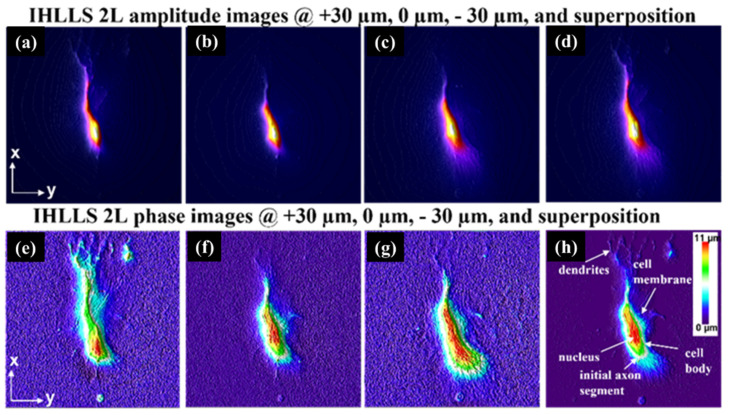
IHLLS 2L imaging of a lamprey spinal cord ventral horn neuron with dendrites; (**a**) Amplitude reconstruction of a neuronal cell at three z-galvo positions: (**a**) +30 µm, (**b**) 0 µm, (**c**) −30 µm, and (**d**) the superposition of all three; Phase reconstruction of a neuronal cell at z-galvo positions: (**e**) +30 µm, (**f**) 0 µm, (**g**) −30 µm, and (**h**) the superposition of all three. (Images are taken from [41] with permission).

To determine the characteristics of the system for biological imaging we labeled neurons (ventral horn neurons from lamprey spinal cord) with a fluorescent dye (Alexa 488 hydrazide, ThermoFisher). Data from beyond the maximum area (78 × 78 μm) are not visible either using the original LLS or using IHLLS 1L imaging when the z-galvo mirror and the z-piezo stage objective were moved simultaneously. Similar results as the LLS were obtained by using the IHLLS 2L (Figure 25a–d) from a maximum projection of just three reconstructed amplitude images from IHLLS holograms at zgalvo = ±30 μm, and 0 μm, but in a larger visible sample area. The IHLLS 2L approach can be used to investigate the structure and function of cells and tissue by allowing access to reconstructed phase information (Figure 25e–h).

By combining two lenses with focal lengths, fd1 and fd2 mm, we obtained a reconstruction distance above and below the reference plane (microscope objective position or the middle of the FOV of the camera) corresponding to controllable sample reconstruction depths up to 40 μm and −40 μm, and a total of 80 μm volume height. From this, a 3D perspective representation of the quantitative phase contrast image of a neuron is displayed in Figure 25e–h. The pixels encode quantitative values of the optical path length (OPL) within the fluorescently labeled neurons. This includes the neuron’s soma and its subcellular structures such as axon and dendrites. The scale in Figure 25h, right) compares the OPL (from LUT) to neuronal morphological depth (in µm).

### 10.2. Current and Future Challenges

Although we eliminated the z-piezo objective motion needed to maintain the object focus, there are a few challenges in approaching this work. The conventional LLS system uses a very low dose of light and low exposure time without a polarizer in the detection path. Our approach can improve rate of acquisition data for reconstruction of 3D structures and enables phase imaging of these structures. However, emission intensity in IHLLS is necessarily reduced by a required polarizer. Nevertheless, using digital incoherent holography with the double diffractive lens, FINCH technique, and randomly selected pixels requires only one polarizer mounted before the SLM, to align the input beam to its active axis limiting light loss. Moreover, the objective z-axis position is fixed, which results in brighter images at the center of the z galvo range. Therefore, either the excitation light intensity, or the exposure time, or both, must be increased from the middle of the z-galvo scanning range toward the two ends of the scanning range to maintain a similar signal efficiency as in the conventional LLS. These also must be low enough to preserve the biological samples. Nevertheless, the LLS enables fluorescent imaging with a minimal baseline excitation intensity on which to base this approach allowing minimal photodamage and bleaching. The SLM was also positioned at deflection angle of 11°. This was the lowest possible angle that generated active beam steering at the highest efficiency while allowing positioning of the telescope lenses. Another challenge in this configuration is the beam size of 17.6 mm in the back focal plane of the microscope objective (Nikon Apo LWD 25 × 1.1 W (Bricktown, NJ, USA)), which is too large for the SLM chip size (17.6 mm × 10.6 mm, Meadowlark Optics, Longmont, CO, USA). To work around this, we decided to place the SLM in a double 4f optic system configuration made by two lenses of 200 mm focal lengths and two lenses of 125 mm focal lengths. This combination has also helped to decrease the $z_{h_{min}}$ to about 664 mm.

### 10.3. Advances in Science and Technology to Meet Challenges

We have demonstrated the capacity of the IHLLS system to reconstruct 3D positions of beads using a very reduced number of z-galvo mirror scanning planes [41] when compared to the original LLS system using the dithered mode. We also showed the same or slightly improved resolution of the bead positions. The IHLLS system can therefore enhance the rates that can be achieved for volumetric and multiplane imaging. This will enable faster probing of three-dimensional morphology of biological samples as well as dynamical changes in this morphology.

The use of IHLLS can enhance lattice light sheet microscopy in several ways. In LLS focus of the emission objective must be maintained in the focal plane of the lattice sheet, requiring movement of a bulky objective, which takes time, but also at the high speeds of LLS imaging will apply fluid movements and pressure fluctuations across the sample. This will potentially distort images, but in addition will provide noise to transient measurements of cell surfaces caused by rapid activity, for example action potential firing in excitable cells. We have demonstrated that IHLLS can be used to resolve artificially applied changes in neuron shape. We will further our studies to relate these changes to physiological activity.

### 10.4. Conclusions

IHLLS is a promising technique which can enable tomographic imaging using LLS excitation at a maximum detector FOV of $208\;\mu m^{2}$, by giving access to the complex amplitude of emission light. In addition, IHLLS can achieve faster volumetric acquisition and multiplane imaging to probe dynamical changes in three-dimensional morphology and signaling within biological samples. Axial resolutions can be improved by capturing images at multiple z-galvo steps prior to reconstructing them. This minimizes the hologram reconstruction increment, δz, to improve localization of sample points. We conclude that this optical and mechanical design of an incoherent imaging system provides expanded function to the existing lattice light-sheet system and can potentially open new imaging modalities in this as well as other light sheet imaging instruments. The IHLLS optical path is readily added as an accessory or can be simply applied as an add-on feature.

## 11. Multiwavelength-Multiplexed Incoherent Digital Holography Based on Computational Coherent Superposition (Tatsuki Tahara, Ayumi Ishii, Takako Koujin, Atsushi Matsuda, Yuichi Kozawa, and Ryutaro Oi)

### 11.1. Status

Multidimensional image sensing is one of the actively researched themes in both science and industry. Multidimensional information, such as 3D images, multiple wavelengths, and polarization distributions, is important to observe realistic scenes of remote locations, structures in nanoscopic and microscopic fields of view, and electromagnetic wave distributions in all wavelength bands. The acquisition of 3D information is important, particularly when a person or a machine perceives and observes 3D structures of samples. Color and polarization information is useful for identifying and distinguishing objects. Ordinarily, multiple image sensors, color filters, and an array of polarizers are used to obtain multidimensional information.

Holography [118,119] is a 3D information sensing technique utilizing light interference. A 3D image of an object is recorded as a 2D interference fringe image. Furthermore, not only a 3D image but also wavelength and polarization information can be recorded simultaneously on a 2D image. Holography exploits phase encoding in the recording of multidimensional information simultaneously on multiplexed hologram(s) and decoding in the reconstruction of object waves at multiple wavelengths and polarization directions separately on the spatial [120] and temporal [121] frequency domains or the computational coherent superposition (CCS) scheme [122,123,124]. Moreover, even spatially and temporally incoherent light can be recorded as a hologram by incoherent holography [1,7,125,126,127].

CCS is based on phase-shifting interferometry (PSI) and regarded as multiwavelength-multiplexed PSI. CCS can be merged into any optical system that adopts PSI and, therefore, CCS and Fresnel incoherent correlation holography (FINCH) [1,7] can be combined to conduct the multiwavelength 3D imaging of incoherent light. FINCH is a spatially incoherent digital holography technique and adopts PSI to remove undesired-order diffraction waves from the recorded holograms. The main contributions of FINCH include the high-quality 3D imaging of incoherent light by applying PSI to incoherent holography and improvements of optical transfer function (OTF) and point spread function (PSF) in incoherent imaging [8,31,85]. By combining CCS and FINCH, it is possible to conduct full-color 3D imaging with an improved PSF [42]. In this chapter, we introduce experimental results with a CCS-FINCH system.

#### CCS-FINCH

**Figure 26:**
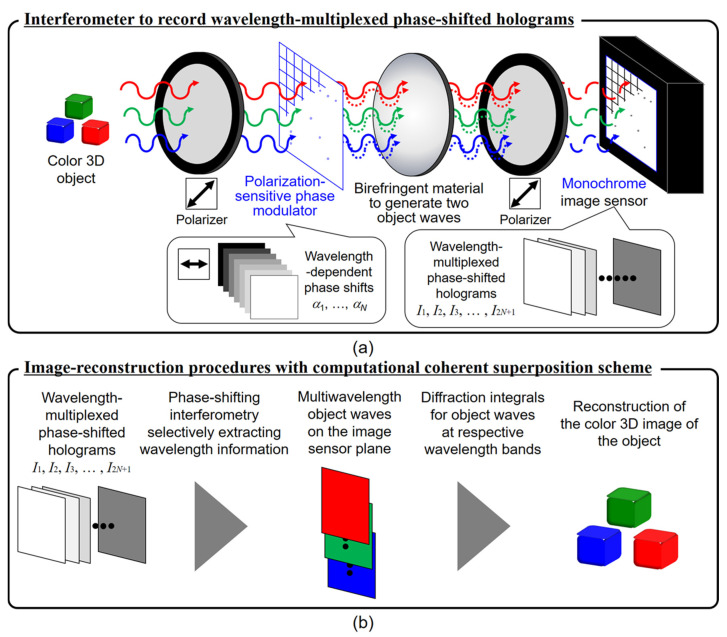
Schematic of CCS. (**a**) A self-interference digital holography system with CCS and FINCH. (**b**) Image-reconstruction procedures.

Figure 26 illustrates the schematic of CCS [122,123,124]. CCS can be incorporated in a self-interference phase-shifting digital holography system. Figure 26a shows an optical system that includes a CCS scheme and polarization-based FINCH system. Multiwavelength incoherent light waves are diffracted from a color 3D object whose polarization is aligned by a linear polarizer. Using a polarization-sensitive phase modulator, phase shifts are introduced to the horizontally polarized component in the object wave. Here, the phase shifts are dependent on the wavelength and, therefore, different phase shifts are introduced to different wavelengths of the object wave. Then, a birefringent material modulates horizontally and vertically polarized components in the object wave. Different modulations are added to respective components and then two object waves with different wavefront curvature radii are generated. Another polarizer aligns the polarization directions of the object waves, and the two waves interfere with each other. The difference between the wavefront curvature radii for a point of an object generates a Gabor zone plate (GZP) pattern. An object is regarded as a summation of object points and, therefore, the incoherent summation of GZP patterns forms a spatially incoherent hologram. A monochrome image sensor records a spatially incoherent hologram digitally. By introducing wavelength-dependent phase shifts with a phase modulator, a monochrome image sensor records wavelength-multiplexed phase-shifted digital holograms. In the image-reconstruction procedures, we apply a signal-processing algorithm of CCS [124] to the recorded holograms. In an algorithm, PSI is applied to the recorded holograms to selectively extract wavelength information and multiwavelength object waves are separately retrieved. Then, diffraction integrals are calculated with object waves at respective wavelengths and the color 3D image of the object is reconstructed.

**Figure 27:**
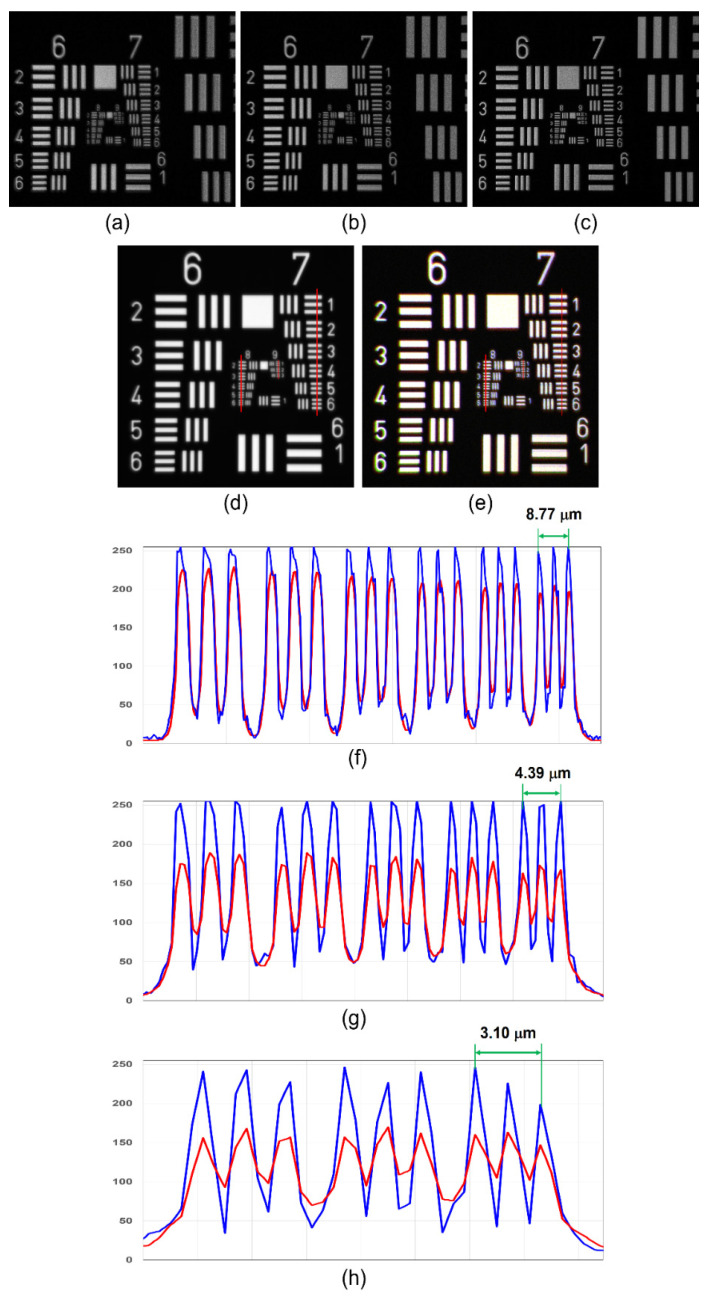
Experimental results. Reconstructed images at wavelengths of (**a**) 618, (**b**) 530, and (**c**) 455 nm. (**d**) Photograph of the specimen and (**e**) color-synthesized image generated from (**a**–**c**). Plots of borderlines in (**d**,**e**) along groups (**f**) 7, (**g**) 8, and (**h**) 9. Red lines in (**d**,**e**) indicate the locations where the plots are selected. In (**e**), the white balance was calculated during synthesis. Red and blue lines in (**f**–**h**) indicate the results obtained from (**d**,**e**), respectively.

We conducted an experiment to show full-color 3D imaging with an improved PSF by using a CCS-FINCH system [42]. The CCS-FINCH system shown in Figure 4 in [42] was used for this experiment. A halogen lamp set in a commercially available microscope (Olympus, IX-73) was used as the light source and a USAF1951 test target was set as the specimen to evaluate the PSF. The experimental conditions were the same as those in [42]. For comparison, a focused 2D image of the specimen was recorded using the same system after setting the transmission axis of the polarizer as the vertical direction so as not to generate self-interference or a digital hologram. Figure 27 shows the experimental results. In an ordinary incoherent imaging system, the PSF degrades with increasing spatial frequency of the specimen. However, using FINCH, the PSF was improved in comparison with an incoherent 2D imaging system. Furthermore, using CCS, multiwavelength imaging with seven wavelength-multiplexed phase-shifted images was successfully conducted. Thus, full-color 3D imaging with an improved PSF was experimentally demonstrated.

### 11.2. Other Remarks and Conclusions

Color 3D imaging with FINCH was initially performed with temporal division in 2007 [24]. Then, CCS-FINCH enabled wavelength-multiplexed recording with a small number of holograms. By combining CCS-FINCH and single-shot phase-shifting holography [13,33,57,127,128], the single-shot multicolor 3D imaging of incoherent light was realized [129] partly at the cost of a reduced space–bandwidth product. When using a phase spatial light modulator to display a Fresnel phase lens into CCS-FINCH, wavelength separation was well performed for RGB-LED light [130] but multiple object images were generated at undesired wavelengths owing to the wavelength dependence of the Fresnel lens. In the implementation, it is effective to combine CCS with COACH [29] to obtain a color 3D image without higher-order diffraction images. In COACH, wavelength information is separated utilizing both the difference between PSFs at different wavelengths and correlation calculations [131]. The correlation-based method is suitable for single-shot imaging with a wide space–bandwidth product at the cost of a slightly reduced wavelength resolution, and the interference-based method with CCS has the ability to perform multicolor 3D imaging with a certain wavelength resolution at the cost of reduced spatial or temporal resolution. Compared with a single-shot implementation with a color image sensor [132], a single-shot CCS-FINCH system [129] obtains a wide space–bandwidth product at respective wavelengths [133] and a high light-use efficiency due to the absence of absorption by a color filter. On the other hand, a color filter increases temporal coherence, and therefore self-interference incoherent digital holography with a color image sensor has successfully obtained full-color hologram(s) even of sunlight and white light generated from a halogen lamp [25,132].

In this chapter, we have introduced full-color 3D imaging with an improved PSF by CCS-FINCH and compared it with related methods. The main contributions of CCS to incoherent holography include multiwavelength incoherent 3D imaging with a smaller number of recordings than Fourier spectroscopy [134,135] applied to incoherent holography. CCS-FINCH has also been successfully applied to fluorescence digital holographic microscopy [26,62]. This multiwavelength-multiplexed incoherent digital holography technique will contribute to various filterless multidimensional image sensing applications.

## Data Availability

Data is contained within the article.
